# Supplementary material for: Disruption of GRIN2B Impairs Differentiation in Human Neurons
Source: Stem Cell Reports. 2018 Jun 21;11(1):183–96. doi: 10.1016/j.stemcr.2018.05.018 (PMC6067152; doi:10.1016/j.stemcr.2018.05.018)

Stem Cell Reports, Volume *11*

Supplemental Information

Disruption of *GRIN2B* Impairs Differentiation in Human Neurons

Scott Bell, Gilles Maussion, Malvin Jefri, Huashan Peng, Jean-Francois Theroux, Heika Silveira, Vincent Soubannier, Hanrong Wu, Peng Hu, Ekaterina Galat, S. Gabriela Torres-Platas, Camille Boudreau-Pinsonneault, Liam A. O'Leary, Vasiliy Galat, Gustavo Turecki, Thomas M. Durcan, Edward A. Fon, Naguib Mechawar, and Carl Ernst

Supplementary Figures

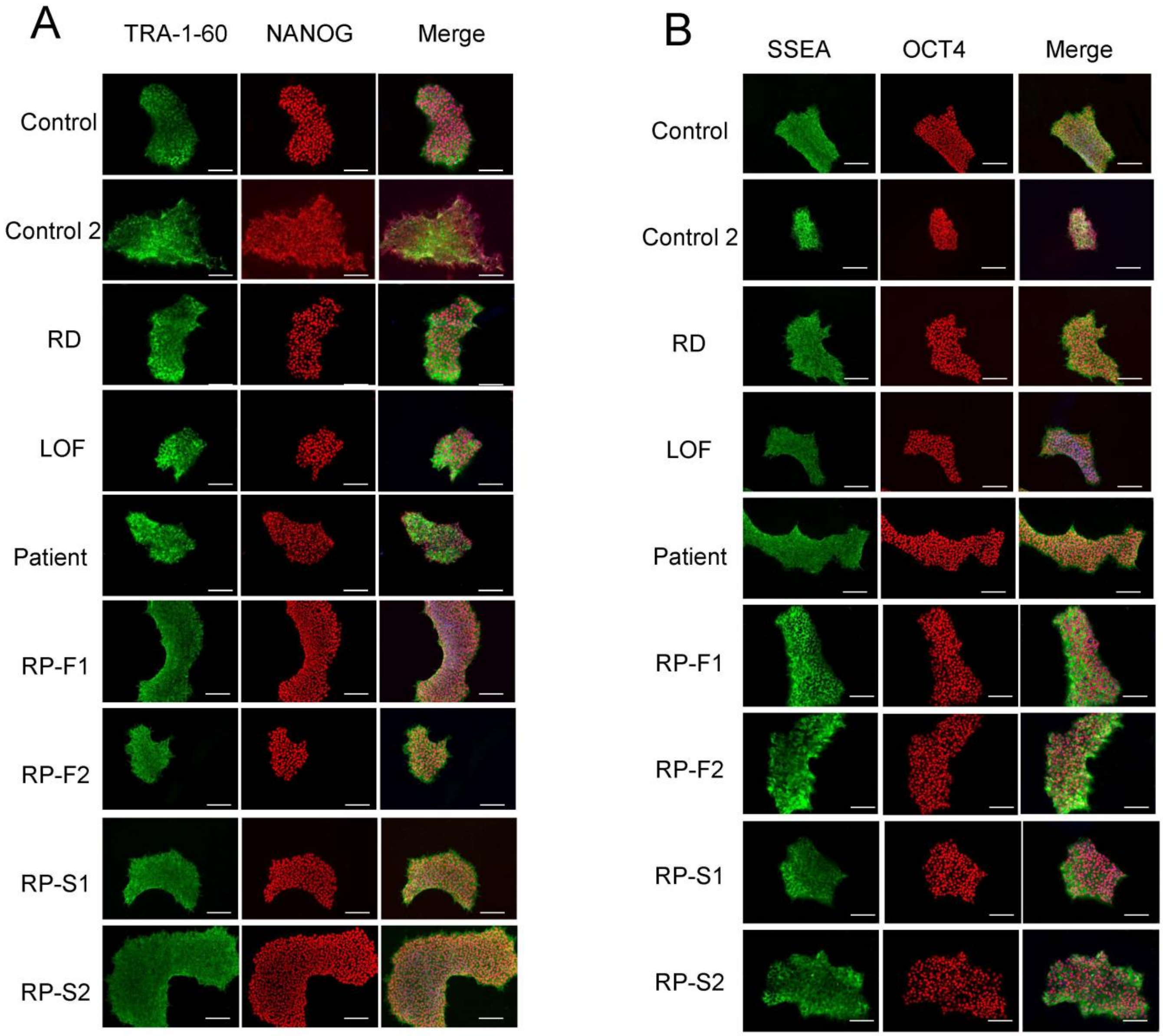

Supplementary Figure 1. Quality Control staining for pluripotency of cell lines used in this study. Related to Figure 1

Staining of the pluripotent markers TRA-1-60 and NANOG (shown in A) and SSEA and OCT4 (shown in B) from all lines used in this study. Scale bar represents 50µm. Select images can be found in Figure 1.

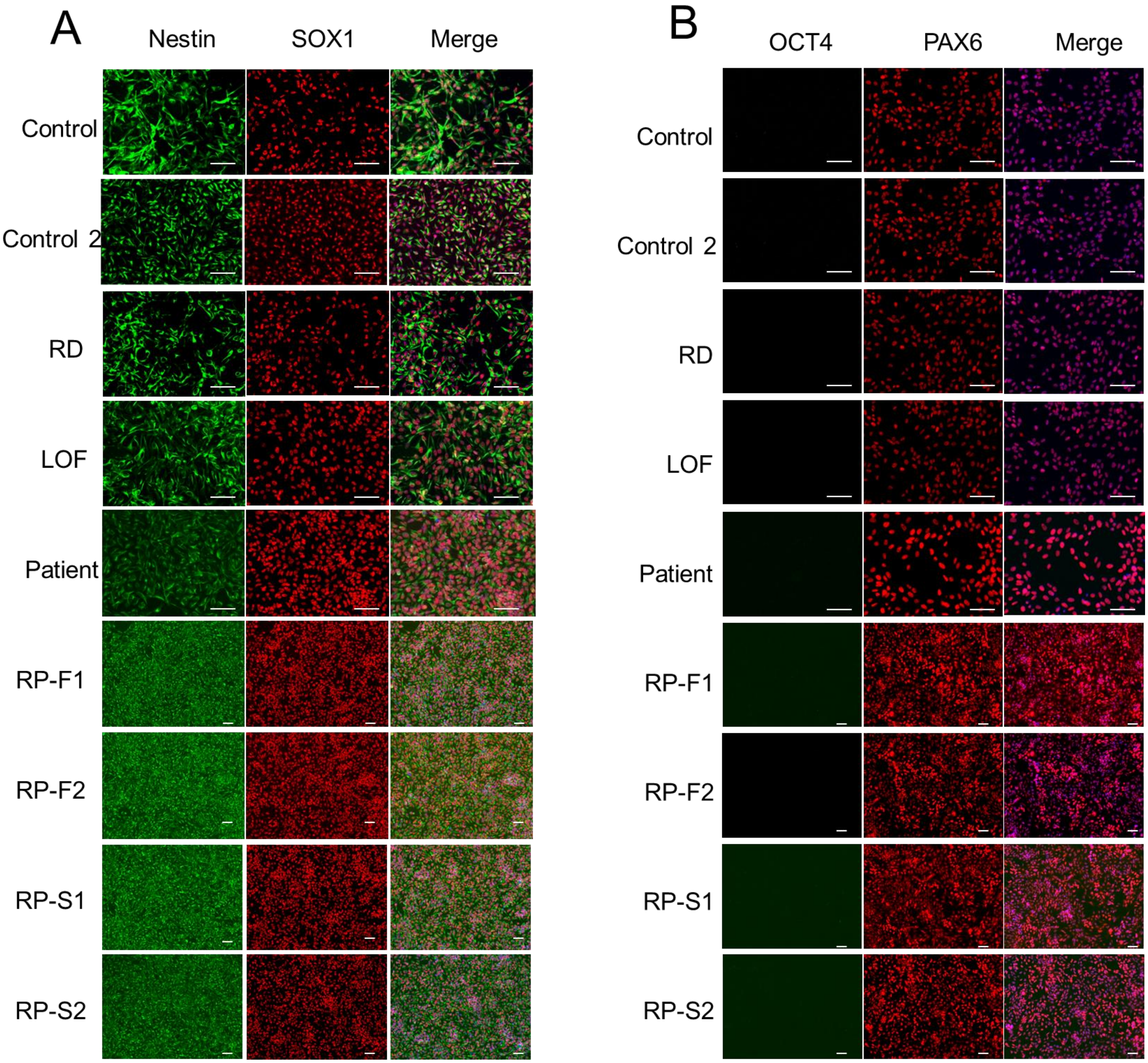

Supplementary Figure 2. Quality Control staining for neural progenitor cells. Related to Figure 1

Immunostaining of Nestin and SOX1 (shown in A), and OCT4 and PAX6 (shown in B) for all cell lines used in this study. Scale bar represents 50µm. Select images can be found in Figure 1. A representative image from A) can be found in Figure 2.

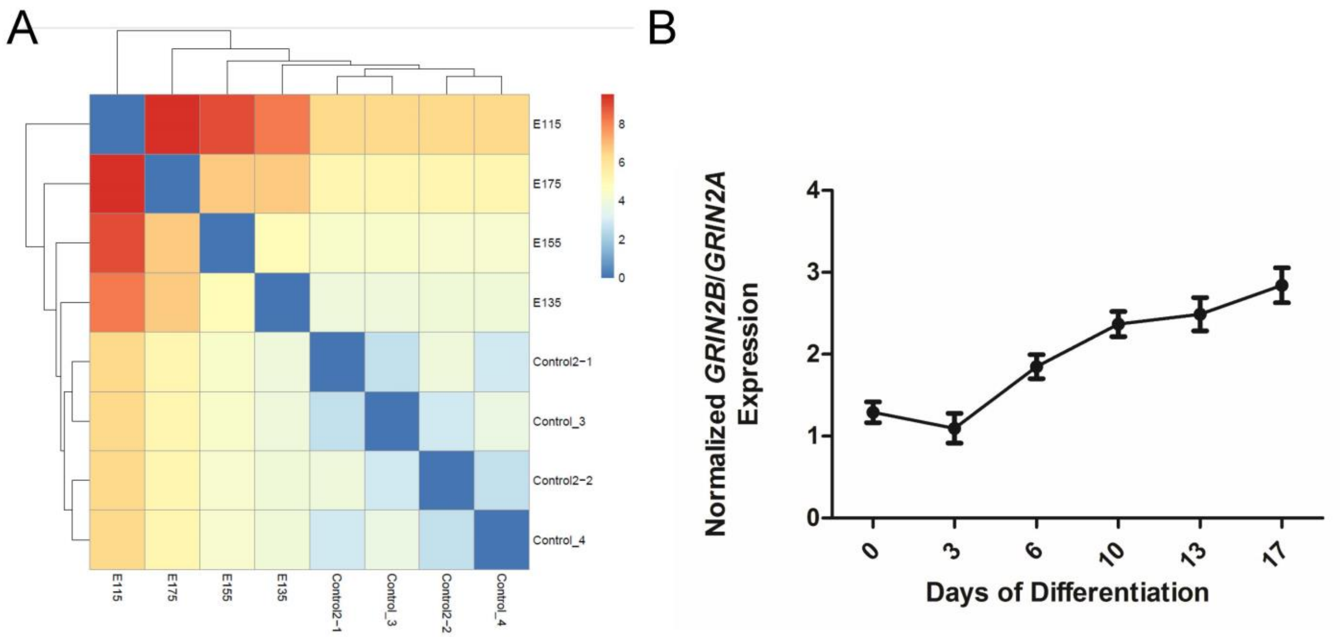

Supplementary Figure 3. Characterization of forebrain neurons. Related to Figure 1

A) Clustering of RNA-Seq data from D30 forebrain neurons with RNA-SEQ data from mouse radial precursor single cell expression profiles at four different timepoints. The Gene matrix was normalized using a regularized log transformation. Mouse radial precursor expression profiles were obtained from Yuzwa et al. (2017).

B) Ratio of GRIN2B/ GRIN2A expression during neuronal development ranging from 0 to 18 days after the initiation of differentiation from NPCs.

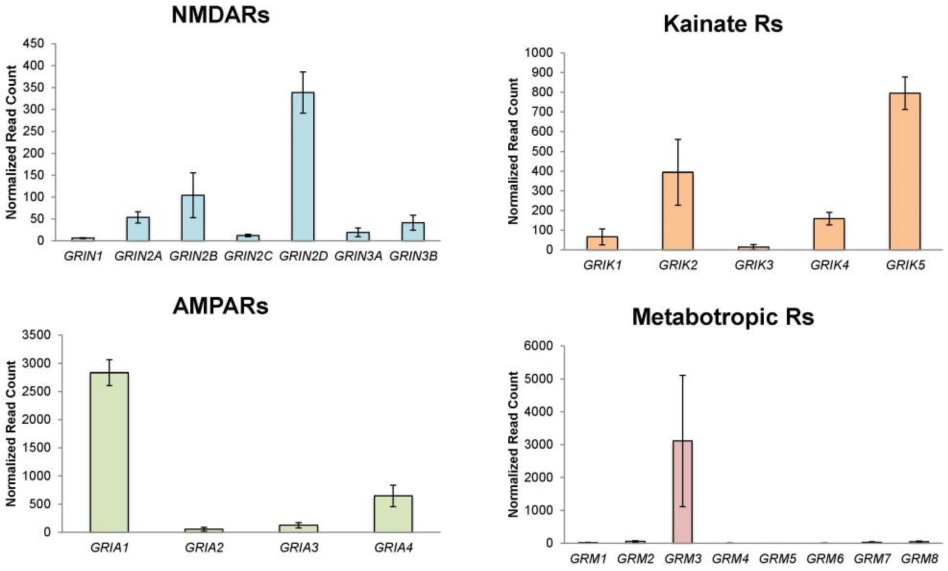

Supplementary Figure 4. Profile of NMDA, AMPA, Kainate, and Metabotropic receptor genes in control NPCs. Related to Figure 2

RNA sequencing reads for all NMDA, AMPA, Kainate and Metabotropic receptor genes. Reads normalized using deseq2 normalization algorithm.

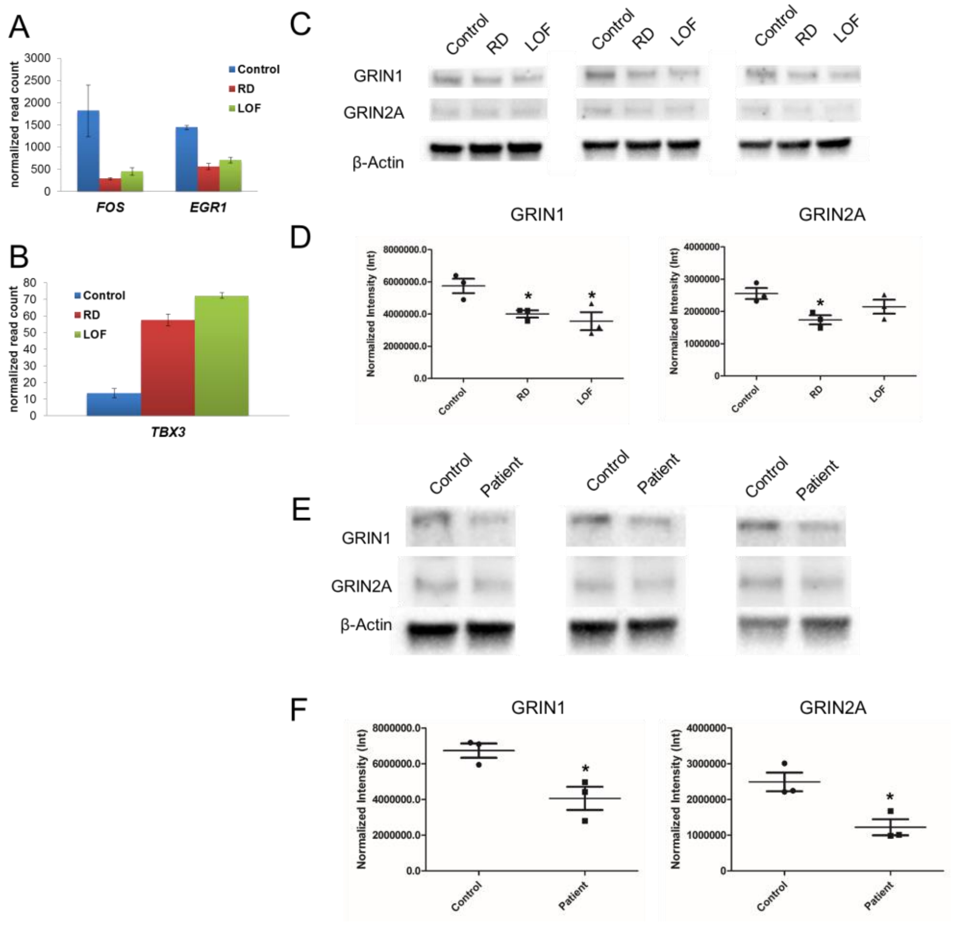

Supplementary Figure 5. Deficiency in *GRIN2B* expression is correlated with decreased expression of *GRIN1* and *GRIN2A*. Related to Figure 3 and Figure 4

A) Immediate early genes FOS and EGR1 show reduced expression in *GRIN2B* deficiency models (RNAseq data), consistent with loss of NMDA signalling

B) Increased TBX3 expression in GRIN2B deficiency models is consistent with cells in a more proliferative state.

C) Independent triplicate Western blots of GRIN1, GRIN2A and β-Actin using lysates from control, RD, and LOF neurons taken after four weeks of differentiation from NPCs (Day=30).

D) Quantification of the Western blots shown in A for GRIN1 and GRIN2A, using β-Actin for normalization. *: $p<0.05$

E) Independent, triplicate Western blots of GRIN1, GRIN2A and β-Actin using lysates from patient and control neurons taken at D=28.

F) Quantification of the Western blots shown in B for GRIN1 and GRIN2A, using β-Actin for normalization. *: $p<0.05$

Supplementary Tables

Supplemental Table 1. Cell lines used in this study. Name, source of the cell line, the sex, age, ethnicity characteristics, reprogramming method and gene editing is listed for each line. Related to Figure 1

Name	Source	Sex	Age	Ethnicity	Clinical Characteristics	Reprogramming Method	Gene Editing Method
Control 1	Coriell (GM07492)	M	17	Caucasian	Healthy	Episomal	N/A
Control 2	Patient Biopsy	M	21	Caucasian	Healthy	Episomal	N/A
Patient	Patient Biopsy	F	5	Caucasian	Delayed development, intellectual disability, hypotonia	Episomal	N/A
RD	Control 1	N/A	N/A	N/A	N/A	Episomal	CRISPR/ $CAS9^{wt}$
LOF	Control 1	N/A	N/A	N/A	N/A	Episomal	CRISPR/ $CAS9^{wt}$
RP-F1	Patient	N/A	N/A	N/A	N/A	Episomal	CRISPR/ $CAS9^{D10A}$ Nickase
RP-F2	Patient	N/A	N/A	N/A	N/A	Episomal	CRISPR/ $CAS9^{D10A}$ Nickase
RP-S1	Patient	N/A	N/A	N/A	N/A	Episomal	CRISPR/ $CAS9^{D10A}$ Nickase
RP-S2	Patient	N/A	N/A	N/A	N/A	Episomal	CRISPR/ $CAS9^{D10A}$ Nickase

Supplemental Table 2: Cell lines and replicates used in this study. The cell lines and number of replicates used in each Figure is listed. Related to Experimental Procedures

Experimental Figure	Cell lines Used	Replicates (n)
Figure 1E	Control 1	8
Figure 2G	Control 1	≥46
Figure 3G	Control 1, LOF, RD	3
Figure 3I	Control 1, LOF, RD	8
Figure 4D	Control 1, Control 2, Patient	3
Figure 4F	Control 1, Patient	7
Figure 4K	RP-F1, RP-F2, RP-S1, RP-S2	6
Figure 5C	Control 1	7
Figure 6B	Control 1, RD, LOF, Patient	≥58
Supplementary Figure 3B	Control 2	3
Supplementary Figure 4	Control 1	
Supplementary Figure 5A	Control 1, LOF, RD	3
Supplementary Figure 5B	Control 1, LOF, RD	3
Supplementary Figure 5D	Control 1, LOF, RD	3
Supplementary Figure 5F	Control 1, Patient	3

Supplemental Experimental Procedures

Sanger sequencing

DNA was extracted from iPSCs using a QIAamp DNA Mini Kit (QIAGEN). Amplification of putative CRISPR KO and repair colonies was performed in 25 µl reaction volume consisting of 10 µL nuclease free water, 12.5 µL Taq green master mix, 0.5 µL forward primer, 0.5 µL reverse primer, 0.5 µL DMSO, 1µL template. PCR was performed using a S1000™ Thermal Cycler (BioRad). PCR products were examined by electrophoresis at 100 V for 30 min in a 1.5% (w/v) agarose gel in 1 x TAE buffer to confirm product purity, and then shipped to Genome Quebec (Montreal, Canada) and sequenced on a using a 3730xl DNA Analyzer (Illumina). Primer sequences used to confirm gene editing can be found in CRISPR supplementary materials

RNA extraction and quality control

Cells were washed with PBS and detached using 0.05% trypsin-EDTA, and resuspended in Qiazol (Qiagen). RNA was extracted using an miRNeasy kit (Qiagen). Prior to RNA sequencing, appropriate RNA concentration, 260/230 and 260/280 ratios were determined by using a NanoDrop 2000 UV-Vis Spectrophotometer (Nanodrop). The quality of the RNA and the 28S/18S ratios were assessed using an Agilent 2100 Bio analyser (Aligent) and the RNA 6000 NanoChip (Aligent). All samples with RIN values below 9 were excluded from further analysis.

RNA-Sequencing

All libraries were prepared by expert technicians at the McGill University and Genome Quebec Innovation Center. Replicates for each cell line were grown in different T75 flasks, and extraction of RNA was done independently for each flask. Eight libraries were run per lane of an Illumina HiSeqV4 2500 flow cell (125 bp paired-end reads), which achieved an average of ~40 million reads per library. For bioinformatic processing, we used FASTX-Toolkit, TopHat Bowtie2, and Cufflinks2 with default parameters to preprocess, align, and assemble reads into transcripts, estimate abundance, and test differential expression.

Comparison of transcriptomics profiles

Mouse radial precursor single cell expression profiles at three different timepoints (embryonic days 11, 13, 15 and 17) were obtained from Yuzwa et al. (2017). Average expression profiles were computed on cortical cells for each developmental stage. In an effort to compare our RNASeq read counts with their Single Cell transcript counts, we divided each gene's read counts by its transcript's size. We selected a number of common genes present in our RNASeq and their 4 timepoints. Correspondence between our human genes and their mouse genes was established based on gene

symbols. The resulting dataset is composed of 4 average expression profiles (one for each timepoint) and 4 RNASeq Control samples from our experiment (composed of two batches of two samples) over a total of 11869 genes. The gene matrix was normalized using a regularized log transformation in DESeq2. Limma's removeBatchEffect algorithm was applied to the data to reduce the impact of the 3 expected batches (data from Yuzwa et al. and the two batches from our controls). Batch-corrected log transformed counts were then used for two hierarchical clustering experiments, respectively using distance and Pearson's R as dissimilarity/similarity metrics. Both raw and analyszed data were uploaded to the GEO database (https://www.ncbi.nlm.nih.gov/geo/) under the study number GSE114685.

GEO analysis

Genes were considered as signicantly differentially expressed if they displayed a Bonferroni corrected P-value below 0.05. Differentially expressed genes were analysed using DAVID annotation tools (https://david-d.ncifcrf.gov/). To determine functional enrichments in our sets of significant differentially expressed genes, the first three out five layers of annotations were selected from Gene Expression Omnibus classifications in the categories “biological process”, “cellular component” and “molecular function” A significant enrichment was considered in those categories for values below 0.05 after correction using a Bonferroni method.

Tables for Supplemental Experimental Procedures

Primers and probes used for qPCR analysis. Related to Experimental Procedures

Gene target	Reference
GAPDH	4310884E (Applied Biosystems)
GRIN2B	Hs00168230_m1 (Applied Biosystems)
GAPDH	Hs.PT.39a.22214836 (IDT)
MET	Hs.PT.58.339430 (IDT)
MKI67	Hs.PT.58.27920212 (IDT)

Antibodies used in immunocytochemistry. Concentration used, Supplier and Catalog number is provided for each antibody. Related to Experimental Procedures

Antibody	Concentration Used	Supplier	Catalog Number
Tuj1	1/2000	Abcam	ab14545
Nestin	1/2000	Stemcell Technologies	60091
SOX1	1/1000	Stemcell Technologies	60095
OCT4	1/100	Stemcell	60093

		Technologies	
PAX6	1/500	Stemcell Technologies	60094
TRA-1-60	1/100	Abcam	ab109884
Nanog	1/100	Abcam	ab109884
SSEA	1/100	Abcam	ab109884
VGLUT1	1/300	Abcam	ab77822
GABA	1/500	Abcam	ab86186
GFAP	1/500	Abcam	ab7260
S100B	1/200	Abcam	ab52642
MAP2	1/100	Abcam	ab109884
GRIN2B	1/250	Abcam	ab93610
KI67	1/500	Abcam	ab92742
MET	1/100	Abcam	ab51067
ALEXA 488	1/2000	Invitrogen	A-11008
ALEXA 555	1/2000	Invitrogen	A-21422

Antibodies used in western blotting. Concentration used, supplier, and catalog number is provided for each antibody. Related to Experimental Procedures

Protein Target	Concentration Used	Supplier	Catalog Number
GRIN2B	1/2000	Abcam	ab65783
GRIN2A	1/1000	Abcam	ab124913
GRIN1	1/1000	Abcam	ab109182
KI67	1/3000	Abcam	ab92742
MET	1/1000	Abcam	ab51067
β-actin	1/5000	Abcam	ab8227
C-FOS	1/1000	Abcam	ab190289
P-CREB (S133)	1/1000	Abcam	ab32096
CREB	1/1000	Abcam	ab32515
Rabbit Anti-Mouse IgG		Abcam	ab97046
Goat Anti-Rabbit IgG	1/5000	Abcam	ab6721

Supplementary Information about *GRIN2B* KO CRISPR Experiment (Related to Figure 3)

Section of *GRIN2B* Gene targeted: hg19_dna range=chr12:13722703-13722942

TGATGTTTTGGACTGGCCATCAGTAGAGGACAAATGGGCACTTTCCCTTTCTTGAA
CTCACCATCTCCAAAGAGCTGCAGGATAGCAAGGTCCACCTGGCGCTTCCACCCA
GAATCTTTTTGGATGGCAATGCCATAGCCAGTGGAAGCAAAGACCTTCCCACTGCC
AATGGTCACCAGCTTGCAGCCTTCATCTCTGCCTGCCATATAGTTCAGCACTGCTG
CATCATAGATGAAGGCA

FWD CRIPSR gRNA sequence:

GATGGCAATGCCATAGCCAGTGG

REV CRISPR gRNA sequence:

GGACCGCGAAGGTGGGTCTTAGA

Sanger Forward Primer sequence: TTTGGACGTGGCCATCAGTAG

Sanger Reverse Primer sequence: TATATGGCAGGCAGAGATGAAG

Gel of PCR products for Sanger Sequencing

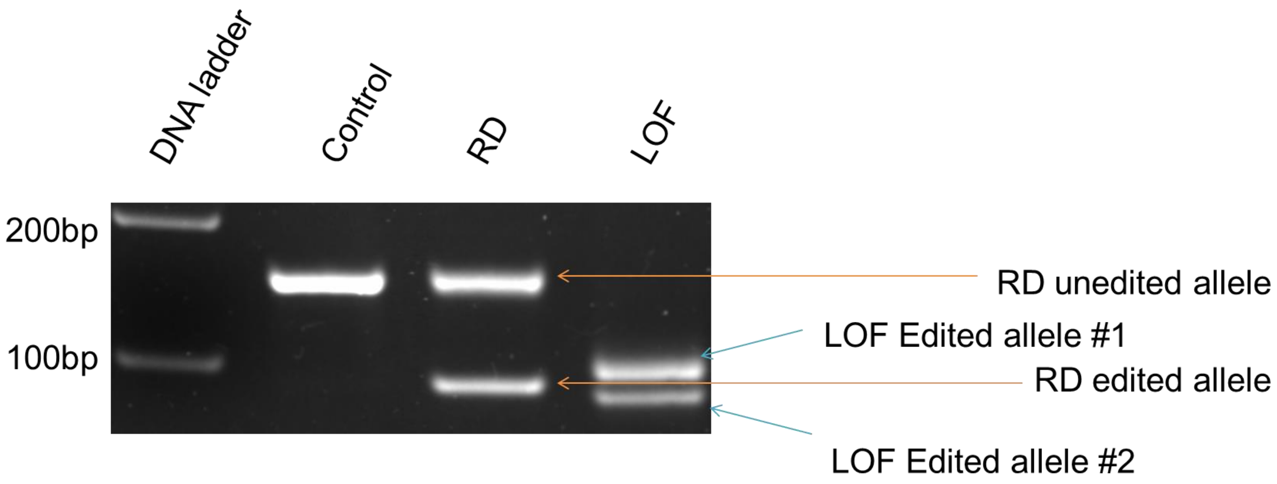

Fasta sequences from Sanger sequencing

Control

ttctgaagaagtgagttttggatggcctcagtagaggacaaatgggcactttccctttcttgaactcaccatctccaaagagctgcaggatagcaaggtccacctggcgcttccacccagaatctttttggatggcaatgccatagccagtggaagcaaagaccttcccactgccaatggtcaccagcttgcagccttcatctctgcctgccatatagaa

RD High Band

ttctgaagaaggtgagttttggatggccatcagtagaggacaaatgggcactttccctttcttgaactcaccatctccaaagaagctgcagggatagcaaggtccacctggcagctgtcccactgccaatggtcaccagcttgcagccgtcatctctacctgccatatagaa

RD Low Band

Nngggaagaagtgagttttggatggccatcagtagaggaaaatgggcactttccctttcttgaactcaccatctccaaagagctgcaggatagcaagaccttcccactgccaatggtcaccagcttgcagccttcatctctgcctgccatatagaa

LOF High Band

ttctgaaagagttgagttttggatggccatagtagaggacaaatgggcactttccctttcttgaactcaccatctccaaagagctgcaggatagcaaggtccacctggcgcttccacccagaatctttttggatggcaatgccatagccagtggaagcaaagaccttcccactgccaatggtcaccagcttgcagccttcatctctgcctgccatatagaa

LOF Low Band

cctggagaaggtgagttttggatggccatcagtagaggaaaatgggcactttccctttcttgaactcaccatctccaaagagctgcaggatagcaaggtccacctgccaatggtcaccagcttgcagccttcatctctgcctgccatatagaa

Alignment

Control	caaggtccacctggcgcttccacccagaatctttttggatggcaatgccatagccagtggaagcaaagaccttcccactgcca
RD High Band	caaggtccacctggcgcttccacccagaatctttttggatggcaatgccatagccagtggaagcaaagaccttcccactgcca
RD Low Band	caag--tccaaacatca
LOF High Band	caag--accttcccactgcca
LOF Low Band	caaggtccacctg---cca

Chromatograms

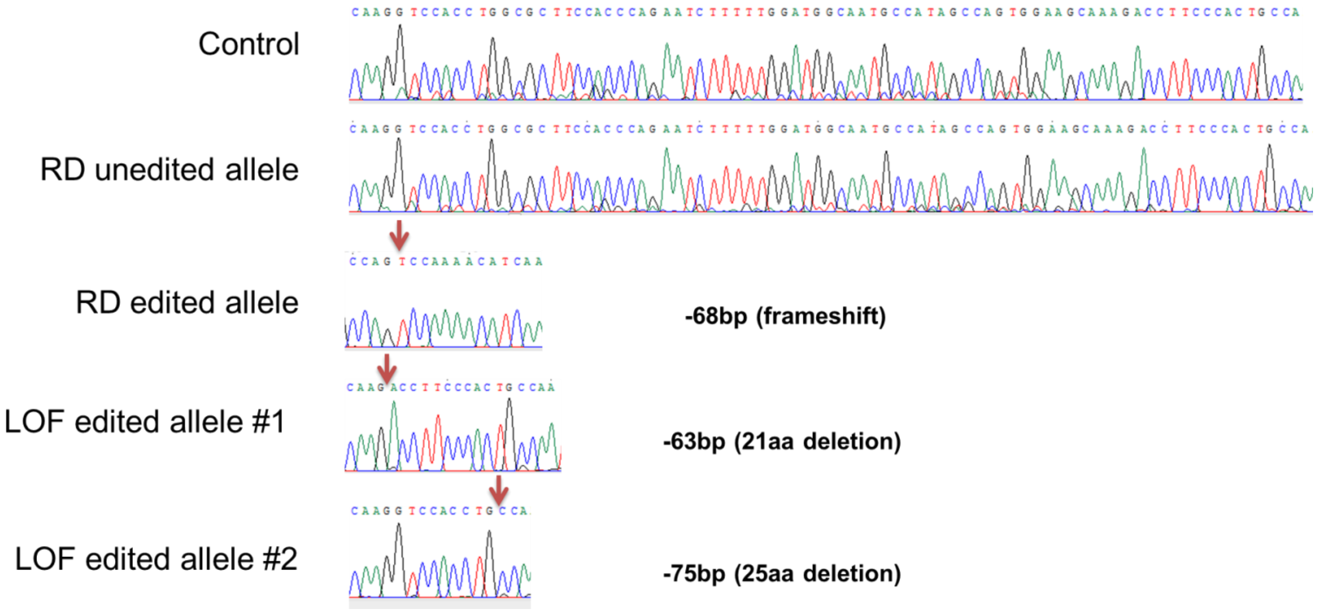

Supplementary Information about *GRIN2B* Repair CRISPR Experiment (related to Figure 4)

Point mutation sequence: (NM_000834.3:c.1238A>G)

Allele ID:152907

Variant type:single nucleotide variant

Cytogenetic location:12p13.1

Protein change: E413G

Patient Sequence: >hg19_dna range=chr12:13769238-13769704

TTTTGAGGGGAGGAATTTGTCCTTCAGTGAAGATGGCTACCAGATGCACCCGAAAC
TGGTGATAATTCTTCTGAACAAGGAGAGGAAGTGGAAAGGGTGGGGAAGTGGAA
AGACAAGTCCCTGCAGATGAAGTACTATGTGTGGCCCCGAATGTGTCCAGAGACT
GAAGAGCAGGAGGATGACCATCTGAGCATTGTGACCCTGGAGGGGCACCATTTG
TCATTGTGGAAAGTGTGGACCCTCTGAGTGGAACCTGCATGAGGAACACAGTCCC
CTGCCAAAAACGCATAGTCACTGAGAATAAAACAGACGAGGAGCCGGGTTACATC
AAAAAATGCTGCAAGGGGTTCTGTATTGACATCCTTAAGAAAATTTCTAAATCTGTG
AAGTTCACCTATGACCTTTACCTGGTTACCAATGGCAA

FWD CRISPR gRNA sequence: GAAAGTGTGGACCCTCTGAGTGG

REV CRISPR gRNA sequence: TGACAAATGGTGCCCCCTCCAGG

Repair Template FWD:
AATGTGTCCAGAGACTGAAGAGCAGGAGGATGACCATCTGAGCATTGTGACCCTGGAGGAGGCACCATTTGTCATTGTGGAAAGTGTGGACCCTCTGAGTGGAACCTGCATGAGGAACACAGTCCCCTGCCAAAAACGCATAGTCACTGAGA

Sanger Forward Primer sequence: GGGGAAGTGGAAAGACAAGT

Sanger Reverse Primer sequence: CAAAGCTGACTCTCCCATGC

Gel of PCR products for Sanger Sequencing

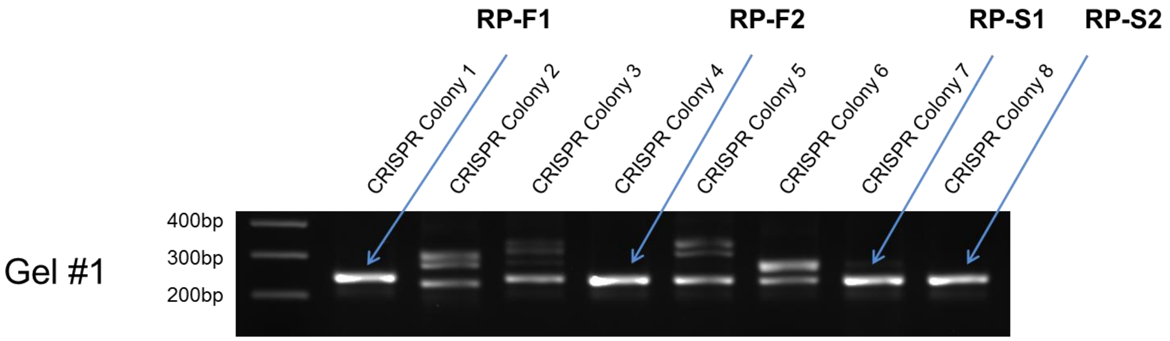

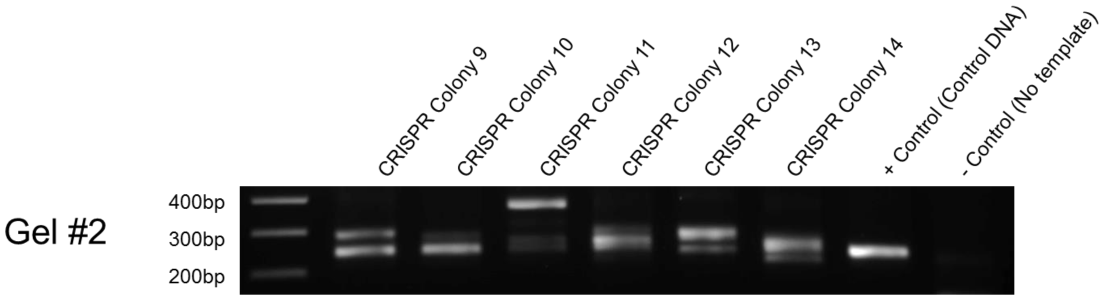

Fasta sequences from Sanger sequencing

Control

tggagagcctatcagtgactatcgtttttggcaggggactgtgttcctcatgcaggttccactcagagggtccacactttccacaatgacaaatggtgcctcctccagggtcacaatgctcagatggtcatcctcctgctcttcagtctctggacacattcggggccacacatagtacttcatctgcagggacttgtctttccacttccccaa

Patient

tggagagcctatcagtgactatcgtttttggcaggggactgtgttcctcatgcaggttccactcagagggtccacactttccacaatgacaaatggtgcctcctccagggtcacaatgctcagatggtcatcctcctgctcttcagtctctggacacattcggggccacacatagtacttcatctgcagggacttgtctttccacttccccaa

RP-F1

ggagagcctatcagtgactatcgtttttggcaggggactgtgttcctcatgcaggttccactcagagggtccacactttccac
aatgacaaatggtgcctcctccagggtcacaatgctcagatggtcatcctcctgctcttcagtctctggacacattcggggcc
acacatagtacttcatctgcagggacttgtctttccacttccccaa

RP-F2

ttggagagcctatcagtgactatcgtttttggcaggggactgtgttcctcatgcaggttccactcagagggtccacactttcca
caatgacaaatggtgcctcctccagggtcacaatgctcagatggtcatcctcctgctcttcagtctctggacacattcggggc
cacacatagtacttcatctgcagggacttgtctttccacttccccaa

RP-S1

tggagagcctaacagtgactatcgtttttggcaggggactgtgttcctcatgcaggttccactcagagggtccacactttcca
caatgacaaatggtgcctcctccagggtcacaatgctcagatggtcatcctcctgctcttcagtctctggacacattcggggc
cacacatagtacttcatctgcagggacttgtctttccacttccccaaa

RP-S2

Ttggagagcatatcagtgacatgcgtttttggcaggggactgtgttcctcatgcaggttccactcagagggtccacactttcc
acaatgacaaatggtgcctcctccagggtcacaatgctcagatggtcatcctcctgctcttcagtctctggacacattcgggg
ccacacatagtacttcatctgcagggacttgtctttccacttccccaa

Alignment (on reverse strand)

Control	CCACTCAGAGGGTCCACACTTTCCACAATGACAAATGGTGCCTCCTCCAGGGTCACAATGCTCAGATGGTCATCCT
Patient	CCACTCAGAGGGTCCACACTTTCCACAATGACAAATGGTGCCACCTCCAGGGTCACAATGCTCAGATGGTCATCCT
RP-F1	CCACTCAGAGGGTCCACACTTTCCACAATGACAAATGGTGCCACCTCCAGGGTCACAATGCTCAGATGGTCATCCT
RP-F2	CCACTCAGAGGGTCCACACTTTCCACAATGACAAATGGTGCCACCTCCAGGGTCACAATGCTCAGATGGTCATCCT
RP-S1	CCACTCAGAGGGTCCACACTTTCCACAATGACAAATGGTGCCTCCTCCAGGGTCACAATGCTCAGATGGTCATCCT
RP-S2	CCACTCAGAGGGTCCACACTTTCCACAATGACAAATGGTGCCTCCTCCAGGGTCACAATGCTCAGATGGTCATCCT

Chromatograms (on reverse strand)

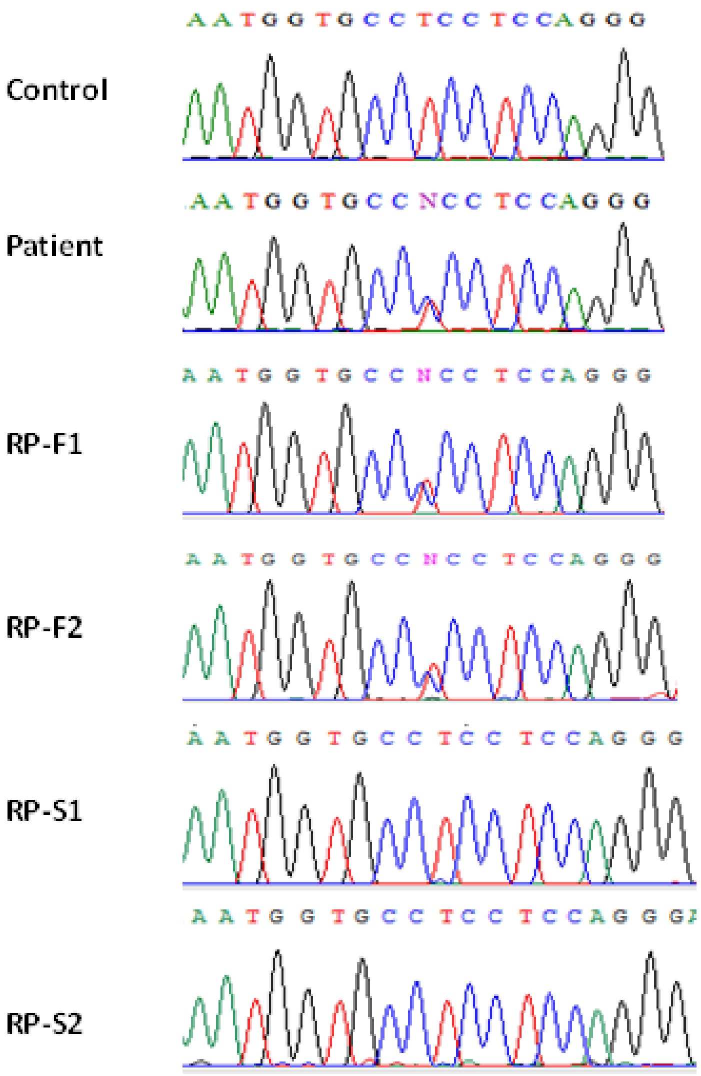

Supplement: Document S1. Supplemental Experimental Procedures, Figures S1–S5, and Tables S1 and S2 [file mmc1.pdf]
