# Supplementary material for: Disruption of GRIN2B Impairs Differentiation in Human Neurons
Source: Stem Cell Reports. 2018 Jun 21;11(1):183–96. doi: 10.1016/j.stemcr.2018.05.018 (PMC6067152; doi:10.1016/j.stemcr.2018.05.018)

Stem Cell Reports
Article
ISSCR
OPEN ACCESS

Disruption of *GRIN2B* Impairs Differentiation in Human Neurons

Scott Bell,[1,4] Gilles Maussion,[1,4] Malvin Jefri,[1] Huashan Peng,[1] Jean-Francois Theroux,[1] Heika Silveira,[1] Vincent Soubannier,[2] Hanrong Wu,[1] Peng Hu,[1] Ekaterina Galat,[3] S. Gabriela Torres-Platas,[1] Camille Boudreau-Pinsonneault,[1] Liam A. O'Leary,[1] Vasiliy Galat,[3] Gustavo Turecki,[1] Thomas M. Durcan,[2] Edward A. Fon,[2] Naguib Mechawar,[1] and Carl Ernst[1,*]

[1]McGill University and Douglas Hospital Research Institute, Department of Psychiatry, 6875 LaSalle Boulevard, Frank Common Building, Room 2101.2, Verdun, Montreal, QC H4H 1R3, Canada

[2]Montreal Neurological Institute, Department of Neurology and Neurosurgery, Montreal, QC H3A 2B4, Canada

[3]Department of Pediatrics, Developmental Biology Program, Stanley Manne Children's Research Institute, Ann and Robert H. Lurie Children's Hospital of Chicago, Northwestern University, Feinberg School of Medicine, Chicago, IL 60611, USA

[4]Co-first author

*Correspondence: carl.ernst@mcgill.ca

https://doi.org/10.1016/j.stemcr.2018.05.018

SUMMARY

Heterozygous loss-of-function mutations in *GRIN2B*, a subunit of the NMDA receptor, cause intellectual disability and language impairment. We developed clonal models of *GRIN2B* deletion and loss-of-function mutations in a region coding for the glutamate binding domain in human cells and generated neurons from a patient harboring a missense mutation in the same domain. Transcriptome analysis revealed extensive increases in genes associated with cell proliferation and decreases in genes associated with neuron differentiation, a result supported by extensive protein analyses. Using electrophysiology and calcium imaging, we demonstrate that NMDA receptors are present on neural progenitor cells and that human mutations in *GRIN2B* can impair calcium influx and membrane depolarization even in a presumed undifferentiated cell state, highlighting an important role for non-synaptic NMDA receptors. It may be this function, in part, which underlies the neurological disease observed in patients with *GRIN2B* mutations.

INTRODUCTION

N-Methyl-D-aspartic acid receptors (NMDARs) are widely expressed in neurons and are composed of different subunits that form specific types of functional glutamate receptors. NMDARs are made up of an assortment of four subunits in a combination of two dimers (Salussolia et al., 2011; Sheng et al., 1994), where the *GRIN1* subunit is the only essential member and the most genetically distant from other members (Cull-Candy et al., 2001). Subunit composition of NMDARs confers different biophysical properties on NMDARs such as glutamate binding affinities, activation/deactivation kinetics, or ion conductance (Cull-Candy et al., 2001). Subunit expression patterns are often specific to developmental location or time window. For example, inclusion of *GRIN2* subunits A–D varies depending on brain region and developmental time window (Monyer et al., 1994), where *GRIN2B* is present in embryonic NMDARs but is replaced in postnatal NMDARs by *GRIN2A* (Williams et al., 1993). The presence of *GRIN2C* likely occurs only in the cerebellum and after birth, and the presence *GRIN3A* and *GRIN3B* in NMDARs may influence synapse formation (Das et al., 1998). These consistent patterns of *GRIN1–3* expression suggest tight regulatory control and highlight the tuning of NMDARs to signal different effects in a cell.

The development of whole-genome sequencing technologies has allowed for major sequencing efforts of patients with neurodevelopmental disorders, and has underscored the importance of *GRIN2B* in human brain development. Large cohort studies for intellectual disability or autism spectrum disorders both have identified loss-of-function (LOF) mutations in *GRIN2B* that cause a severe neurological phenotype of broad spectrum (Endele et al., 2010; O'Roak et al., 2011), a result supported by several case reports (Dimassi et al., 2013; Freunscht et al., 2013; Hu et al., 2016). Homozygous *Grin2b*-deletion mice die at early postnatal stages due to impaired suckling response and show impaired hippocampal long-term depression (Kutsuwada et al., 1996), while heterozygous mice show reduced expression of *GRIN2B* but survive. Human mutations in *GRIN2B* identified as likely pathogenic lead to LOF of one copy of the gene, a result consistent with a dominant genetic disorder due to either haploinsufficiency (reduced dosage [RD]) or production of a mutant gene product (Hu et al., 2016), causing LOF. Fourteen percent (6/44) of human heterozygous *GRIN2B* mutation cases show gross cortical anomalies (Platzer et al., 2017) as measured by magnetic resonance imaging, while all mouse homozygous *Grin2b* mutants have grossly normal cerebral cortices. The large discrepancy in phenotype between human and mouse *GRIN2B* mutants suggests that the role of *GRIN2B* varies between the species.

While the role of *GRIN2B* in mature synapses, usually within hippocampal circuits, is intensely studied (Bliss and Collingridge, 1993), its role in neurodevelopment,

This is an open access article under the CC BY license (http://creativecommons.org/licenses/by/4.0/).

particularly human brain development, is less well understood. GRIN2B-NMDARs (i.e., those NMDARs that have GRIN2B as a subunit) were initially hypothesized to be important in interpreting early signaling cues in the embryonic environment to guide neuronal differentiation (Cohen and Greenberg, 2008) before synapses form. This idea was supported by several studies from almost three decades ago that suggested that NMDARs may be an important part of neuronal differentiation in cortex, cerebellum, and spinal cord (Balazs et al., 1988; Blanton et al., 1990; Brenneman et al., 1990). NMDA receptors are also critical for subventricular zone neural progenitor migration to the cortex in mouse (Behar et al., 1999), an idea consistent with the importance of NMDA receptors in neural stem cells, an unambiguously non-synaptic developmental time point as cells can still become neurons, astrocytes, or oligodendrocytes. Given the reports of the importance of *GRIN2B* in cell differentiation, we reasoned that mutations in *GRIN2B* in human may lead to a neurodevelopmental disorder not only through its well-known role in synaptic plasticity but through a role in differentiating neural stem cells. To address this question without using animal models of brain development, we elected to use human induced pluripotent stem cells (iPSCs) to generate forebrain neurons.

RESULTS

Forebrain Neural Progenitor Cells Respond to NMDA and Express GRIN1

After extensive quality control including mycoplasma testing, endogenous marker staining, and molecular karyotyping in iPSCs (Figures 1A, 1B, and S1), we generated forebrain neural progenitor cells (NPCs) (Figures 1C and S2). We define NPCs as committed forebrain progenitors that cycle indefinitely in basic fibroblast growth factor (bFGF) and epidermal growth factor (EGF) media and have the potential to become astrocytes, oligodendrocytes, or forebrain neurons. When these cells are differentiated for 30 days, >90% of Tuj1-positive cells express glutamatergic (~60%) or GABAergic (~30%) markers, with a fraction (~10%) expressing astrocytic makers (Figures 1D and 1E). As NPCs differentiate, the ratio of *GRIN2B*/*GRIN2A* rises, while cells matured for 30 days have an expression profile closest to mouse subventricular zone radial precursor cells at embryonic day 13.5 (Figure S3). Differentiated forebrain NPCs form clusters as they mature into neurons and express synapsin-1 (Figures 1D and 1E). Differentiated cells are electrically active and demonstrate spontaneous action potentials (Figures 1H–1J).

Studies on subventricular zone NPCs isolated before cortical migration respond to NMDA and express subunits of NMDARs (Behar et al., 1999). To assess human NPCs for the presence of functional NMDARs, we recorded from five independent NPCs (Figures 2A–2C), where two cells showed action potentials and one responded to NMDA, despite universal expression of NPC markers in these cultures (Figure 2D). The GRIN1 and GRIN2B protein was easily identifiable in NPCs via western blot, although expression in NPCs was lower than in these same cells matured for 30 days, as expected (Figure 1E). RNA sequencing of control NPCs (n = 3 cell lines) revealed expression of almost all NMDAR subunits as well as subunits from AMPA, kainate, and metabotropic glutamate receptors (Figure S4). To unambiguously show NMDA response in NPC cultures, we have provided videos (Videos S1 and S2) and images (Figures 2F and 2G) of calcium influx after application of NMDA in NPCs and D5 neurons, where D5 neurons show an increased response, likely reflecting a more mature stage of development. It is not immediately obvious whether some cells that are presumed to be in an NPC state are in fact differentiated. A further unknown is whether this is a function of *in vitro* techniques or may reflect NPC populations within the human brain.

Engineered Reduced Dosage and LOF Mutations in *GRIN2B* Impair Differentiation of NPCs

Using our simultaneous reprogramming and gene-editing protocol (Bell et al., 2017), we generated clonal RD and LOF *GRIN2B* models. RD cells are heterozygous for a frameshift mutation in exon 11 and have one functional copy of *GRIN2B*, whereas LOF cells have two different *GRIN2B* mutant alleles, both with in-frame deletions of a large segment of the glutamate binding pocket (Figures 3A and 3B). RD has a ~50% decrease in *GRIN2B* mRNA expression, whereas LOF has a milder decrease in mRNA expression of ~25% (Figure 3C). Using independent replicates (control n = 4, LOF n = 4, RD n = 2), we differentiated NPCs for 30 days and performed whole transcriptomic sequencing in RNA extracted from these neurons, and found excellent segregation of expression patterns (Figure 3D). Both models of *GRIN2B* deficiency had several genome-wide significant gene-expression differences compared with the isogenic control cells, although we focused on those genes that showed expression differences in both *GRIN2B* mutant models, which revealed 657 differentially expressed genes common to both models (hypergeometric $p < 1.8 \times 10^{-204}$) (Figure 3F). The strongest gene ontology terms for these common 657 genes were associated with genes related to increased cell proliferation and decreased cell differentiation (Figure 3G).

Activation of NMDARs drives immediate-early gene expression (Bading et al., 1993). Both *FOS* (Xia et al., 1996) and *EGR1* (Vaccarino et al., 1992) are immediate-early genes that are downregulated in *GRIN2B* mutation

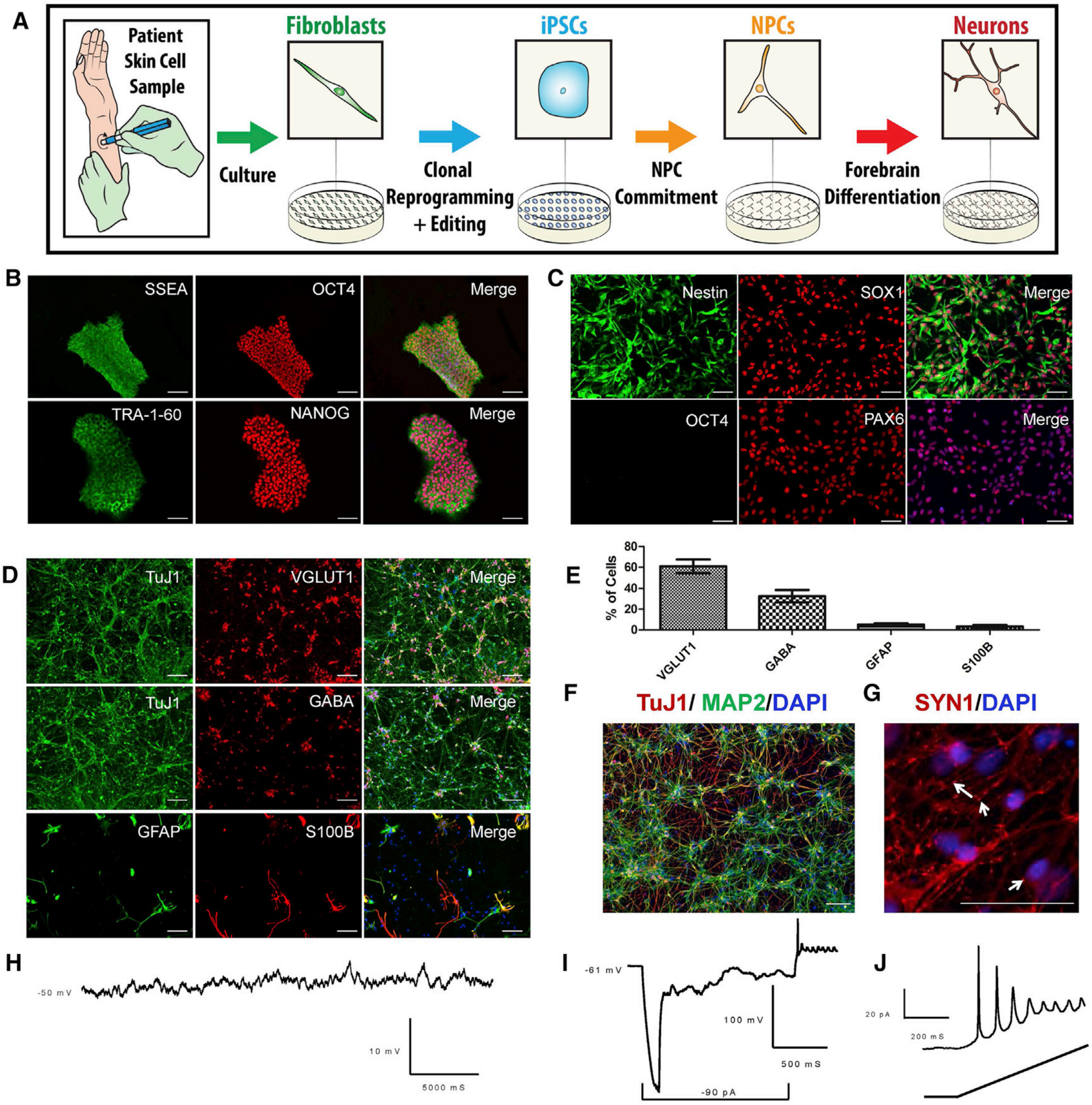

Figure 1. Generation and Characterization of Forebrain Neurons

(A) Outline of procedure used to generate iPSC-derived models of forebrain development.

(B) Representative immunocytochemistry (ICC) for the four key pluripotency markers in control iPSCs. Scale bars represent 100 μm.

(C) Representative ICC of control neural progenitor cells (NPCs) showing the absence of pluripotency markers and the presence of neuronal forebrain markers. Scale bars represent 50 μm.

(D) Representative ICC of forebrain neuronal culture following 30 days of differentiation (D30) from NPCs, demonstrating the relative abundance of glutamatergic, GABAergic, and astrocytic markers in the population. Scale bars represent 50 μm.

(E) Quantification of the percentage of cells positive for markers shown in (D). n = 8 images taken from separate coverslips from the same culture of D30 neurons. Error bars denote SD.

(F) Representative ICC of forebrain neurons differentiated for 30 days from NPCs demonstrating uniform staining for the forebrain marker MAP2. Scale bar represents 50 μm.

(G) Synapsin 1 (SYN1) staining in D30 neurons; Arrows highlight select SYN1 punctate, though many more are visible. Scale bar represents 50 μm.

(H) Representative trace of resting membrane potential (RMP) observed in D18 neurons.

(I) Representative trace of a hyperpolarizing pulses demonstrating that D18 neurons exhibit inward current and spontaneous action potential.

(J) Representative trace of action potentials observed in D18 neurons during current ramp protocol.

See also Figures S1–S3 and Table S1.

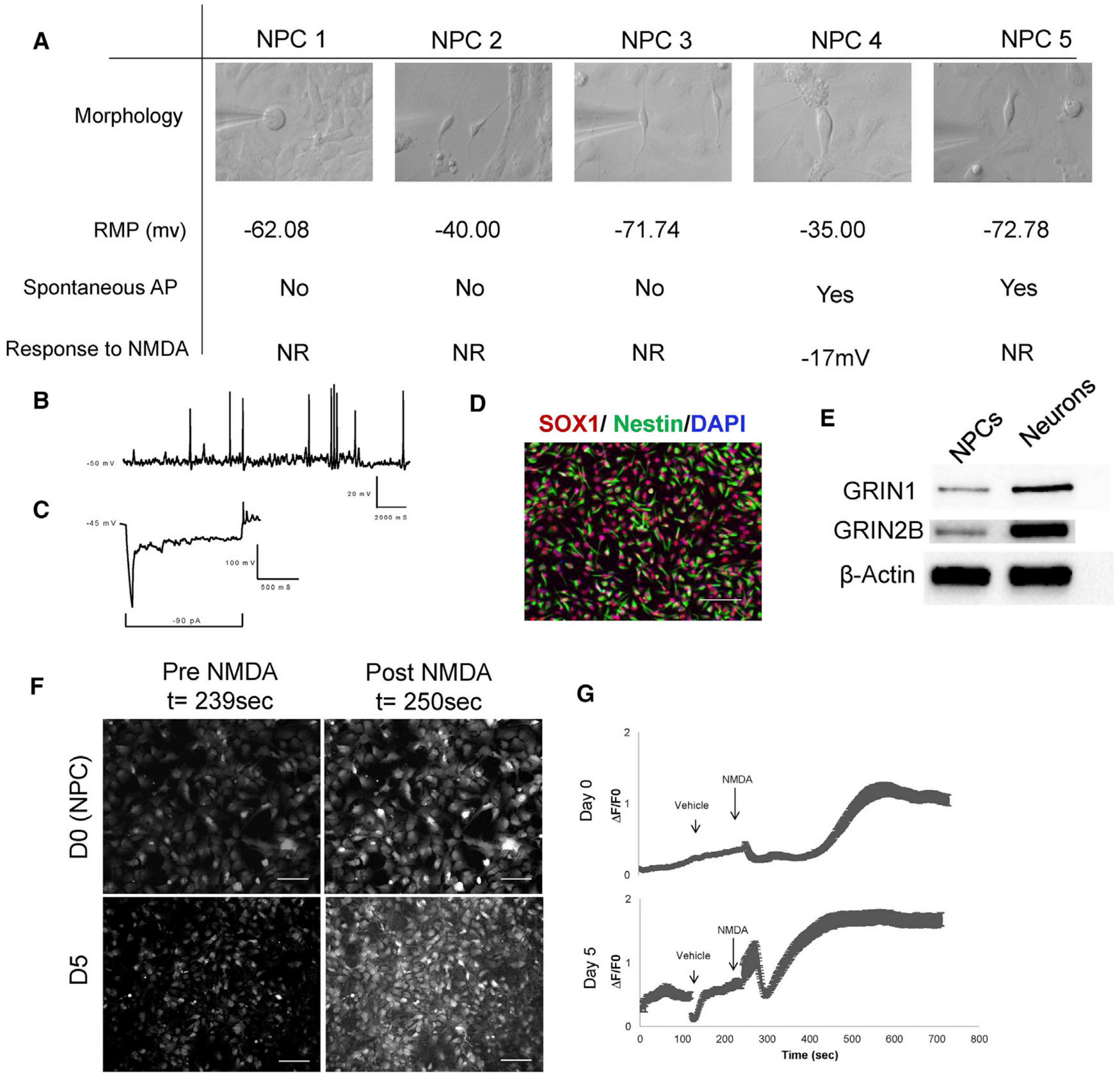

Figure 2. Forebrain Neural Progenitor Cell Cultures Contain a Subpopulation of Cells that Are Electrically Active and Respond to NMDA

(A) Morphology and electrophysiological characteristics of five healthy, control NPCs. Scale bars represent 10 μm.

(B) Trace of RMP obtained from NPC 4.

(C) Representative trace of a hyperpolarizing pulse applied to NPC 4 showing demonstrating inward current and spontaneous action potential.

(D) NPC cells stain uniformly positive for forebrain NPC markers SOX1 and Nestin. Scale bar represents 50 μm.

(E) Western blot showing relative level of expression of GRIN1 and GRIN2B in NPCs and D30 forebrain neurons.

(F) Stills of NPCs and D5 neural cells incubated with the Fluo4 calcium indicator before and after application of NMDA. Stills obtained from Videos S1 and S2. Scale bars represent 40 μm.

(G) Intensity of fluorescent signal detected in NPC and D5 neural cells following application of NMDA and vehicle (DMSO), as shown in Videos S1 and S2. Error bars denote SEM; $n \geq 46$ cells from imaged wells.

See also Figure S4; Videos S1 and S2.

models (Figure S5A). *TBX3*, which itself is sufficient to maintain pluripotency of cells (Russell et al., 2015), is upregulated in *GRIN2B* deficiency models (Figure S5B). We observed decreases in *GRIN1* and *GRIN2A*, although *GRIN2A* was barely detectable (Figures S5C–S5F).

We selected two significant and well-known markers, KI67 and MET, as output measures to assess the differentiation state of neurons and confirm RNA-sequencing data. Assessment of these markers in tandem with *GRIN2B* using qPCR, immunocytochemistry (ICC), and western blot

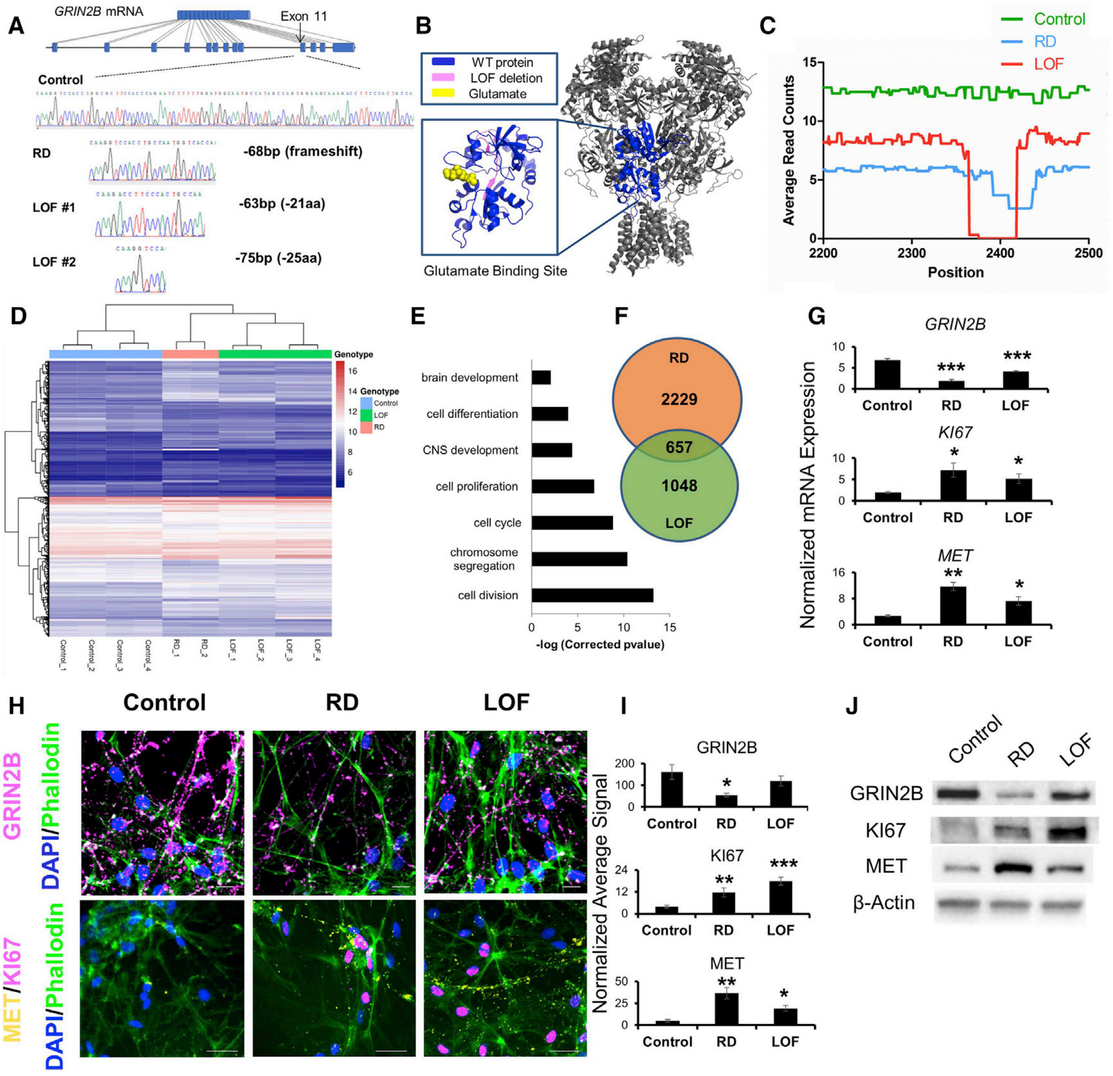

Figure 3. Genetically Engineered *GRIN2B*-Deficient Forebrain Neurons Show Impaired Differentiation

(A) Location of gene-editing site within *GRIN2B*, Sanger sequencing of two edited lines, RD (reduced dosage) and LOF (loss of function). RD is heterozygous with only a single alteration resulting in a frame-shifted protein. LOF has two edited alleles, both of which are in-frame.

(B) Structure of the NMDA receptor, with a magnified view of the glutamate binding site. The region of the glutamate binding site deleted in the LOF model is highlighted in pink.

(C) RNA sequencing reads at the site of editing in transcripts obtained from control, RD, and LOF forebrain D30 neurons after 30 days of differentiation.

(D) Hierarchical clustering of control, RD, and LOF D30 neurons after RNA sequencing. Heatmap of the commonly differentially expressed mRNAs in RD and LOF conditions compared with control.

(E) Gene ontology terms related to significant enrichment of genes commonly deregulated in *GRIN2B* RD and in *GRIN2B* LOF differentiated neurons compared with controls. Corrected p values are expressed as −log.

(F) Venn diagram showing the number of genes exclusively or commonly deregulated in *GRIN2B* LOF and in *GRIN2B* RD differentiated neurons.

(G) Validation of *GRIN2B*, *KI67*, and *MET* mRNA differential expression in LOF and RD D30 neurons by qPCR. mRNA expression is normalized to *GAPDH* expression. Error bars denote SEM; n = 3 independent experiments, with each data point obtained from a separate culture of neuronal cells. *p < 0.05; **p < 0.01; ***p < 0.001.

(H) Representative ICC images of GRIN2B, KI67, and MET immunopositive neurons in control, RD, and LOF conditions. Neurons were fixed at D30 of differentiation. Scale bars represent 50 μm.

(legend continued on next page)

(Figures 3G–3J), showed that while *GRIN2B* was consistently reduced in RD and LOF neurons compared with controls, KI67 and MET were consistently increased. This suggested that LOF and RD neurons were more immature than control neurons differentiated for the same amount of time.

A Missense Mutation in *GRIN2B* Impairs NPC Differentiation and Is Rescued by Genetic Repair

We next generated neurons from a well-studied (Adams et al., 2014) patient with autism and moderate intellectual disability. The subject has a heterozygous mutation (E413G) in the glutamate binding pocket of *GRIN2B* (Figures 4A and 4B), which is reported to decreases glutamate signaling >50-fold (Adams et al., 2014). Assessing the neurons in steps identical to those for the gene-edited *GRIN2B* models, we were able to fully recapitulate the deficient maturational state observed in RD and LOF neurons in patient neurons (Figures 4D–4G).

A pathway whereby CREB becomes phosphorylated at serine 133 after NMDA stimulation and cell maturation has been identified (Sala et al., 2000). To confirm deficiency in this pathway in patients and genetically engineered models of *GRIN2B* deficiency, we performed western blots to determine the protein levels of P133-CREB and CFOS, output markers of NMDA activation albeit non-specific (Xia et al., 1996). The data strongly support the hypothesis that mutant *GRIN2B* impairs NMDA signaling (Figure 4H).

Patient cells can show altered levels of GRIN2B and other output measures due to genetic background. To address this, we corrected the patient mutation back to the wild-type sequence in two clonal lines (Figures 4I and 4J; Supplemental Experimental Procedures). Using clonal cell lines from the patient who failed to repair as control (n = 2), we differentiated all NPC lines for 30 days as assessed output makers GRIN2B, KI67, and MET via qPCR. We observed significantly higher expression of GRIN2B in repaired cells and lower levels of *KI67* and *MET* in failed repair patient cells (Figure 4K).

Pharmacological Block of NMDARs Impairs NPC Differentiation

Loss of *GRIN2B*, and presumably deficient NMDA signaling, increases MET and KI67 while decreasing *GRIN2B* expression. To determine whether pharmacological blockade of NMDARs or GRIN2B phenocopied these effects, we applied two concentrations of 2-amino-5-phosphonovalerate (APV), a competitive antagonist of NMDA, as well as ifenprodil, an uncompetitive inhibitor of NMDARs that contain GRIN2B (Williams, 1993) for 30 days in culture (Figure 5A). We performed protein assessments of GRIN2B, KI67, and MET and found that both APV and ifenprodil produced a decrease in GRIN2B expression, but a significant increase in both KI67 and MET expression (Figures 5B–5D).

Mutations in *GRIN2B* Show Impaired Responses to NMDA

To assess whether there is a functional consequence to both genetically engineered and the patient missense mutation in *GRIN2B*, we differentiated NPCs for 21 days and performed live calcium imaging and electrophysiological recordings. All three *GRIN2B*-deficient cell lines showed a reduced response to NMDA application compared with a control cell line (Figures 6A and 6B; Videos S3–S6). Electrophysiological recordings also presented decreased frequency and amplitude of responses after application of NMDA compared with control cells (Figures 6C and 6D).

DISCUSSION

This work provides a description of iPSC-derived models of *GRIN2B* mutations. All models point to a significant role of *GRIN2B* and NMDARs in cell differentiation, consistent with previous reports showing that stimulation of NMDA receptors affects neuron development (Aamodt and Constantine-Paton, 1999; Blanton et al., 1990; Tovar and Westbrook, 1999). We propose a model whereby GRIN2B-NMDA receptors are critical for signal transduction in neural stem cells. Deficits in this process delay or impair differentiation, including *GRIN2B* expression itself, further impairing differentiation. This suggests a feedforward loop whereby NMDA signaling leads to more expression of *GRIN2B*, and thus more NMDA signaling. We hypothesize that this feedforward loop is not specific to *GRIN2B*, but rather the general differentiation state of the cell. Glutamate signaling through NMDA in neural stem cells or immediately postmitotic neurons may be critical for cells to interpret their environment and differentiate accordingly. In our study we could detect NMDA response in some NPCs as well as action potential generation. There are two possible explanations for this. (1) Cells that stain positive for PAX6, NESTIN,

(I) Quantification of GRIN2B, KI67, and MET signals in control, RD, and LOF D30 neurons. The expression level expressed as normalized average signal is: (mean KI67 or MET pixel intensity) × (number of pixels above threshold/number of DAPI-positive pixels). Error bars denote SEM; n = 3 independent experiments, with each data point representing quantifications of coverslips obtained from separate cultures of each cell line. *$p < 0.05$; **$p < 0.01$; ***$p < 0.001$.
(J) GRIN2B, KI67, and MET western blots of lysates from control, LOF, and RD forebrain neurons at D30.
See also Figure S5.

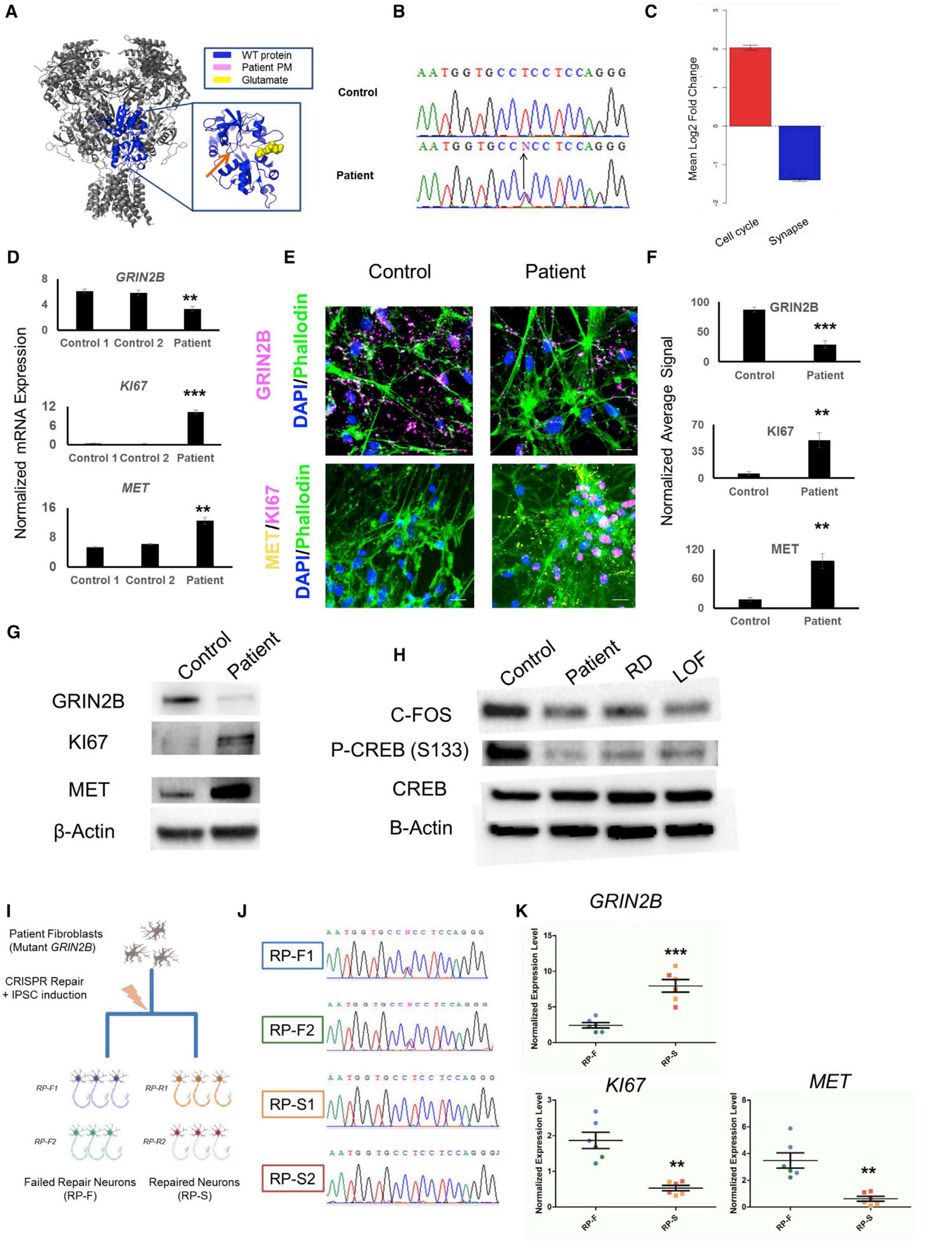

(legend on next page)

and SOX2 are not truly NPCs but rather cells that have differentiated. On close inspection (Figure 2, NPC #4) some NPCs show bipolar morphology, which has implications for these markers in these types of studies. It also has implications for the probabilistic nature of proliferation and differentiation itself, specifically that NPCs may constantly want to differentiate but are held in a proliferative state by the presence of growth factors, with a small minority of cells differentiating regardless under these conditions *in vitro*. (2) Alternatively, NPCs have functional NMDARs that contain *GRIN2B*, in which case the definition of NMDAR function needs to be expanded beyond its role in the synapse and synapse assembly, as has been suggested in mouse (Behar et al., 1999). We favor the latter explanation without discounting the former, but neither may be mutually exclusive.

These data are consistent with a model of neurodevelopmental disease whereby any genetic alteration that alters the precise timing of neuronal differentiation may lead to altered numbers of progenitor cell populations and/or integration of cells into developing circuits (Ernst, 2016). In the current work, loss of *GRIN2B* function may retain cells in a more proliferative-like state, impairing differentiation and, presumably, how neurons integrate into developing circuits. In our view, this is the link between the divergent and extensive list of genes that when mutated lead to variable phenotypes related to intellectual disability. For example, mutations in *PTEN*, *CHD8*, or *CDKL5* all lead to neurodevelopmental disease, and all have a role in cell proliferation.

There have been intensive studies of the role of genes expressed at synapses in neurodevelopmental diseases (Bourgeron, 2015), and we suggest that *GRIN2B* action in neural stem cells may also play a role in these diseases. This leads to a larger question: might other genes with strong associations with neurodevelopment and usually considered in a synaptic context (e.g., *NRXN1* [Kim et al., 2008] or *SHANK3* [Monteiro and Feng, 2017]) also have a role in early cell differentiation? Our study suggests that perhaps other genes considered to have a primarily synaptic function might play a key role in developing neurons.

EXPERIMENTAL PROCEDURES

Somatic Cell Reprogramming

The induction of iPSCs and their subsequent differentiation into neuronal cells was carried out using methods identical to those described previously (Bell et al., 2017). All cell lines were generated from fibroblasts. Control fibroblasts were obtained from the Coriell Cell Repository (Camden, USA), and patient fibroblasts were obtained from the Ann & Robert H. Lurie Children's Hospital of Chicago (Chicago, USA) in adherence with ethical research principles and under protocols approved by the local institutional review board. Further information regarding the cell lines used in this experiment can be found in Table S1.

Fibroblasts were reprogrammed using episomal reprogramming vectors containing Oct4, Sox2, Myc3/4, Klf4, and ShRNA P53 (ALSTEM) and a Neon Transfection System (Invitrogen, Burlington). A total of 5.0×10^5 cells were electroporated and reprogrammed with 5 μg of episomal vectors per reaction.

Figure 4. Forebrain Neurons Derived from a *GRIN2B* Mutation Patient Have Impaired Differentiation that Is Reversible by Genetic Repair

(A) Structure of the NMDA receptor, with a magnified view of the glutamate binding site. The patient mutation E413G is displayed in pink and is highlighted with an orange arrow.

(B) Sanger sequencing of the patient and a healthy control at the site of mutation in *GRIN2B*.

(C) Average fold change of genes differentially expressed in iPSC-derived neurons from patient E413G compared with controls belonging to the Cell Cycle or Synapse GEO terms.

(D) qPCR validation of *GRIN2B*, *KI67*, and *MET* mRNA upregulation in D30 neurons derived from the patient compared with control. Data normalized to *GAPDH* expression. Error bars denote SEM; n = 3 independent experiments, with each data point obtained from a separate culture of neuronal cells: $^{**}p < 0.01$; $^{***}p < 0.001$.

(E) Representative ICC images of GRIN2B, KI67, and MET immunopositive neurons for patient and control D30 neurons. Scale bars represent 25 μm.

(F) Quantification of GRIN2B, MET, and KI67 immunopositive signals in neurons from patient D30 neurons compared with control. Error bars denote SEM; n = 7 independent experiments, with each data point representing quantifications of coverslips obtained from separate cultures of each cell line. $^{**}p < 0.01$; $^{***}p < 0.001$.

(G) Western blot of GRIN2B, KI67, MET, and β-actin using lysates obtained from control and patient D30 forebrain neurons.

(H) Western blot of C-FOS, P-CREB, CREB, and β-actin using lysates obtained from control and patient, RD, and LOF D30 forebrain neurons.

(I) Diagram of the experimental procedure used to generate failed repair (RP-F) and successful repair (RP-S) neurons from patient fibroblasts.

(J) Sanger sequencing of two failed and successful repaired lines at the site of mutation shown in (B).

(K) Normalized expression level of *GRIN2B*, *MET*, and *KI67* mRNA in failed and successful repair D30 neurons. Measurements are matched by color to the specific line to which they correspond (blue: RP-F1; green: RP-F2; orange: RP-S1; red: RP-S2). Error bars denote SEM; n = 6 independent experiments, with each data point obtained from a separate culture of neuronal cells. $^{**}p < 0.01$; $^{***}p < 0.001$.

See also Figure S5.

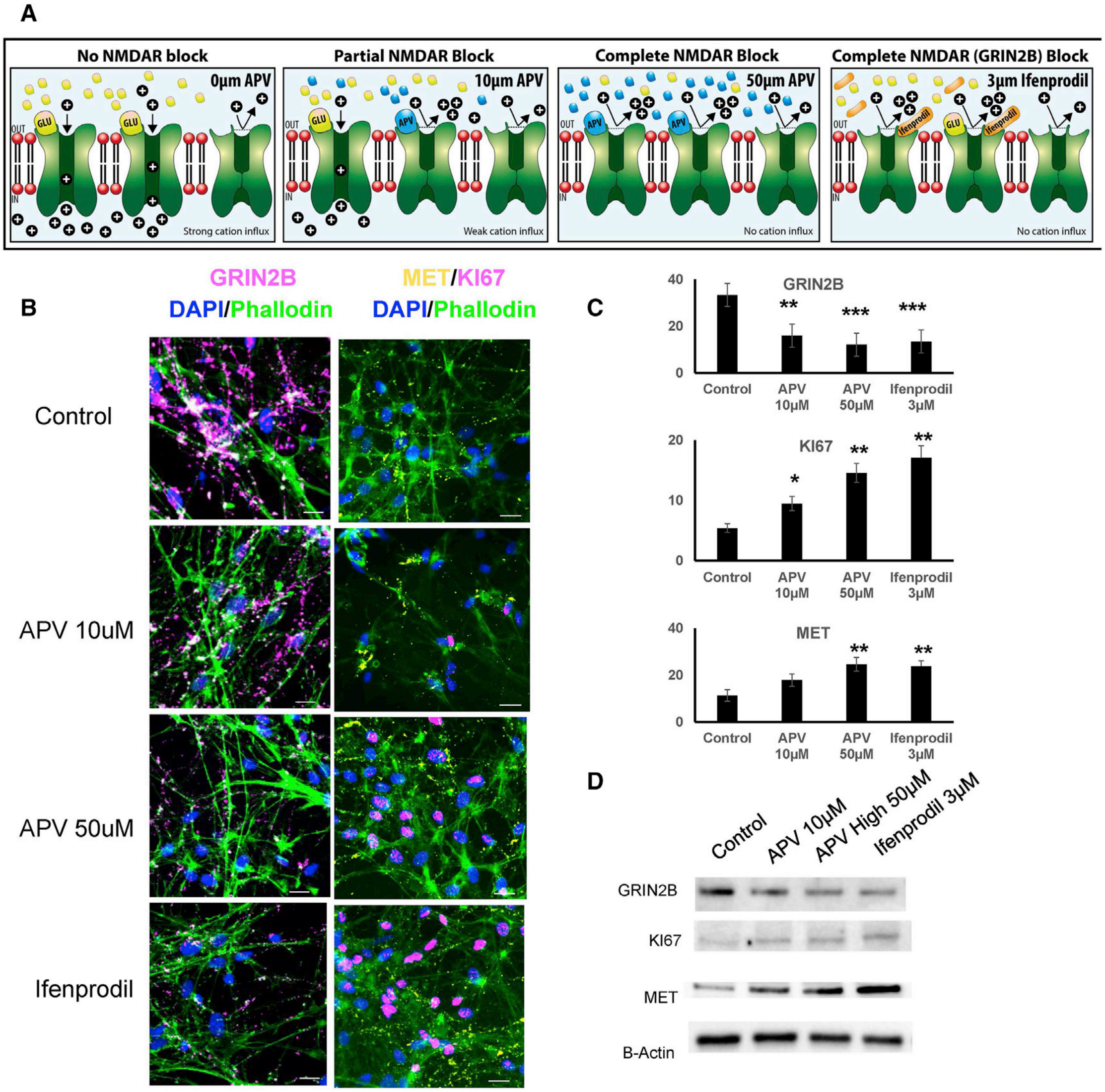

Figure 5. Pharmacological Block of NMDAR Impairs Neuronal Differentiation

(A) Diagram showing the mechanism of action of APV and ifenprodil on NMDAR.

(B) Representative ICC images of GRIN2B; MET, and KI67 immunostaining on D30 control neurons either untreated or treated with APV- or ifenprodil-supplemented medium every 72 hr. Scale bars represent 25 μm.

(C) Quantification of GRIN2B, MET, and KI67 immunopositive signals shown in (B). Error bars denote SEM; n = 7 independent experiments, with each data point representing quantifications of coverslips obtained from separate cultures of each cell line. *p < 0.05; **p < 0.01; ***p < 0.001.

(D) Western blot of GRIN2B, KI67, MET, and β-actin using lysates obtain from control D30 neurons differentiated in APV or ifenprodil-supplemented medium.

See also Figure S5.

Electroporation parameters were as follows: 11,650 V, 10 ms, 3 pulses. Following transfection, cells were plated at extremely low density (~10 cells per well) on tissue culture plates coated with Matrigel (Corning) in 10% fetal bovine serum (FBS) DMEM. The following day, the medium was exchanged for fresh 10% FBS DMEM supplemented with 2 μg/mL puromycin, where applicable (Sigma-Aldrich). Puromycin selection was applied for 48 hr, after which the medium was exchanged with fresh TesR-E7 medium (STEMCELL Technologies, Vancouver). During the induction process, TesR-E7, medium was changed every day. Single iPSC colonies were observed, and could be seen forming from a single skin cell. Once colonies formed a distinct border (~500–1,000 μm in diameter), cells were detached using ReLeSR medium (STEMCELL), and replated in mTesR1 medium (STEMCELL) supplemented with ROCK inhibitor y-27632 (Sigma-Aldrich) at a final concentration of 10 μM.

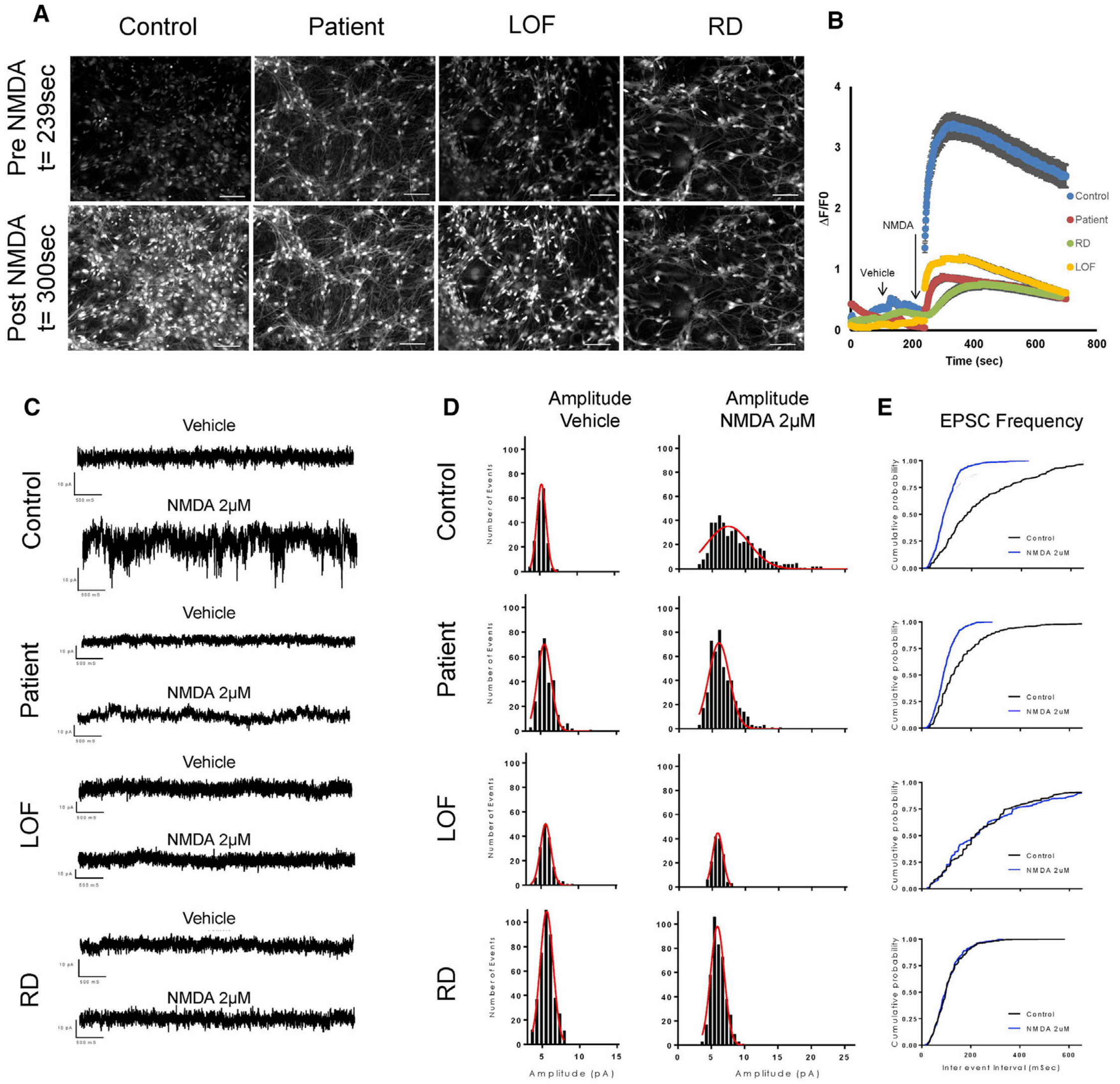

Figure 6. Forebrain Neurons with Genetic Deficiency in *GRIN2B* Show Impaired Responses to NMDA

(A) Stills of D21 control, patient, RD, and LOF forebrain neurons incubated with the Fluo4 calcium indicator before and after application of NMDA. Stills obtained from Videos S3–S6. Scale bars represent 40 μm.

(B) Intensity of fluorescent signal detected in D21 control, patient, RD, and LOF forebrain neurons following application of NMDA and vehicle, as shown in Videos S3–S6. Error bars denote SEM; n ≥ 58 cells imaged from a well containing each cell line.

(C) Frequency of excitatory postsynaptic currents (EPSCs) in control, patient, RD, and LOF neurons after application of vehicle and 2 μM NMDA. Neurons measured between D5 and D9 differentiation time point.

(D) Amplitude histogram distribution of EPSCs after application of vehicle or NMDA as described in (C). Amplitude distribution was fitted using a Gaussian fit.

(E) Frequency of EPSCs after application of vehicle or 2 μM NMDA as described in (C).

See also Figure S5 and Videos S3–S6.

Quality Control of iPSCs

iPSCs were rigorously characterized using several assays. All cells underwent short tandem repeat profiling using ten markers to ensure that derived cells could always be related back to their source cell. All cells were tested for mycoplasma contamination (EZ-PCR Mycoplasma Test Kit [Biological Industries]). Pluripotency was assessed by immunostaining with surface and nuclear pluripotency markers (Figure S1), and spontaneous 7-day embryoid body (EB) differentiation confirmed the capacity to form the three germ layers. Once iPSC lines were stable, we performed array comparative hybridization (aCGH; Cytoscan HD at SickKids Toronto; Thermo Fisher Scientific). No *de novo* CNVs >1 Mb were observed in any colonies, and no *de novo* rare CNVs (<1% in Caucasian population) were observed in genes.

Genetic Engineering

CRISPR gene editing was performed concurrently with iPSC induction, using previously published protocols (Bell et al., 2017). More information regarding CRIPSR design, including the regions of *GRIN2B* targeted, guide RNA sequences, and sequencing chromatograms can be found in Supplemental Experimental Procedures.

iPSC Differentiation to Forebrain Progenitor Cells

iPSCs were dissociated using Gentle Cell Dissociation Reagent (STEMCELL) and resuspended in Neural Induction medium (DMEM/F12 supplemented with N2 [Invitrogen], B27 supplement [Invitrogen], BSA [1 mg/mL], Y27632 [10 μM; AdooQ Bioscience], SB431542 [10 mM; Selleckchem], and noggin [200 ng/mL; GenScript]), onto low-bind plates (Corning) or Petri dishes (Corning). Cells were plated at a density of 2–3 × 10^6 cells per 100-mm^2 plate. Cells were cultured in suspension and monitored for the formation of organoids, which occurred approximately 4 days after suspension. Three days after the formation of EBs, a 70-μm Falcon cell strainer was used to collect aggregations, which were then resuspended in a fresh low-bind/Petri dish in Neural Progenitor (NP) medium (DMEM/F12 supplemented with N2, B27 supplement, bFGF [20 ng/mL; GenScript], EGF [20 ng/mL; GenScript], laminin [1 μg/mL; Sigma-Aldrich]). The medium was exchanged every day for fresh NP medium for 14 days. Following 14 days in NP medium, cell aggregations were resuspended in Final Differentiation (FD) medium (DMEM/F12 supplemented with N2, B27 supplement, brain-derived neurotrophic factor [20 ng/mL; GenScript], glia-derived neurotrophic factor [20 ng/mL; GenScript], laminin [1 μg/mL]). FD medium was changed every 2 days for 7 days. Organoids were plated on polyornithine- and laminin-coated tissue culture plates in Neuron Maturation (NM) medium (DMEM/F12 supplemented with N2, B27 supplement). Following attachment, organoids were dissociated with 0.05% trypsin-EDTA and replated onto fresh polyornithine- and laminin-coated plates in NM medium. Half the medium was exchanged for fresh medium every 3 days.

Videomicroscopy

Cells were seeded in 35-mm MatTek Dishes (MatTek) in the StemDiff Neural Progenitor Medium and differentiated for up to 21 days At the day of the acquisition, the Fluo4 calcium indicator (Thermo Fisher) was incubated for 30 min at a final concentration of 1 μM. Cells were then washed twice for 5 min with the differentiation medium before acquisition. Acquisition were performed using an Axio Observer Z1 microscope (Zeiss) assisted by Zen 2 software. Pictures were collected at every 400 ms for 5 min, with a correction for defined focus every 30 pictures. At picture 120 a vehicle solution was applied, corresponding to cell-culture medium in the case of subsequent NMDA. At picture 240, NMDA was applied at a final concentration of 2 μM. The acquisitions were treated using Fiji/ImageJ software. Threshold was set up to perform a segmentation of the cells, and ROIs were determined and collected through the particle analysis module. Multiple measurement tool from the ROI manager was used to the measure the mean pixel values of each ROI in each picture of the time stack. For the acquisition performed at the NPC stage, a manual segmentation was required to analyze Fluo4 fluorescence variations upon NMDA application. The rest of the data collection process remained the same. Once the data were extracted from the time stack, background was subtracted from every single ROI at every time point. Signal variation is expressed as $\Delta F/F_0$, F_0 being the minimal intensity signal for a given ROI after background subtraction and ΔF being the difference between an intensity signal at a given time point and F0. Amplitude of $\Delta F/F_0$ variations following NMDA applications were monitored in the ROIs and averaged to compare responses between the different conditions. The time stacks were submitted to a JPEG compression at 20 fps to obtain videos.

Electrophysiology

For whole-cell patch-clamp recordings, individual coverslips containing differentiated human iPSC-derived neurons were transferred into a heated recording chamber and continuously perfused (1 mL/min) with BrainPhys Neuronal Medium (STEMCELL) bubbled with a mixture of CO_2 (5%) and O_2 (95%) and maintained at 35°C. Whole-cell patch-clamp recordings were obtained using borosilicate pipettes (3–6 MΩ) filled with intracellular solution that contained the following: 5 in mM HEPES, 2 in mM KCl, 136 in mM potassium gluconate, 5 in mM EGTA, 5 in mM Mg-ATP, 8 in mM creatine phosphate, and 0.35 in mM guanosine triphosphate. The pH was adjusted to 7.27 with KOH, and the osmolarity adjusted with distilled water or concentrated potassium gluconate if needed to between 295 and 298 mOsm with an osmometer (Advanced Instruments). After a recording was completed, we corrected the nominal membrane potential in voltage- and current-clamp recordings for the calculated 10-mV liquid junction potential. All potential values reported reflect this correction. Once whole-cell recording had been established, neurons were routinely held in voltage clamp at −70 mV except when examining changes in the resting membrane potential and rheobase, which was performed in current clamp. Cells were only studied if they exhibited a stable holding current and access resistance for at least 10 min before experimental manipulations. Data were acquired using a Digidata 1550A/Multiclamp 700B (Axon Instruments) and Clampex 10.5 (Molecular Devices). Currents were filtered at 2 kHz and digitized at 20 kHz.

qPCR

Reverse transcriptions were done on the total RNA fraction in order to obtain cDNA in 40-μL volume containing 1 μg of total RNA, 0.5 μg of random primers, 0.5 mM dNTPs, 0.01 M DTT, and 400 U M-MLV RT (Invitrogen). The reactions were performed in a total volume of 20 μL on a 384-well plate using either an Applied Biosystems 7900 HT (Applied Biosystems) or a QuantStudio 6 (Thermo Fisher) PCR machine. For each well, PCR mix included 10 μL of 2× No AmpErase UNG master mix (Applied Biosystems) for Taqman assays or 10 μL of Power SybrGreen PCR Mastermix (Life Technologies), 1 μL of primers/probe mix, 2 μL of cDNA, with H_2O up to 20 μL. Serial dilutions of a mix of cDNA ranging between 0.003052 ng and 50 ng were used to generate a calibration curve for an absolute quantification. Expression levels were given as a ratio between the relative quantities of the gene of interest and the endogenous control. GAPDH was used as internal control for normalization. The normalized expression levels were then

compared between cell lines using ANOVA with a post hoc t test. Further details on the primers used for qPCR can be found in Supplemental Experimental Procedures.

Immunocytochemistry and Microscopy

Cells were plated on glass coverslips coated with Matrigel. Once cells were ready for ICC they were washed with PBS and fixed with 3% paraformaldehyde (Sigma-Aldrich) for 15 min. Samples were permeabilized with 0.5% TX-100 (Sigma-Aldrich) in 0.5% PBS-BSA for 15 min and then blocked in 0.5% PBS-BSA for an additional 15 min. Primary antibodies were added in appropriate dilutions in 0.5% PBS-BSA and added to samples for 30 min. Samples were washed, then 0.5% PBS-BSA containing an appropriate dilution of secondary antibody was added to the samples and incubated for 30 min in the dark. Samples were washed with 0.5% PBS-BSA and visualized on an FV1200 laser scanning microscope (Olympus). Further details on the antibodies used for ICC can be found in Supplemental Experimental Procedures.

Data Acquisition from Images

All ICC images were analyzed using the software ImageJ. Images were converted to an 8-bit mode allowing pixel values in a range between 0 and 256. A threshold was set for each channel to discriminate specific signal intensities from the background. The threshold was determined on the condition presenting the highest signal-to-noise ratio. The average pixel intensities above threshold were normalized by the number of DAPI-positive pixels to minimize biases generated by differences in cell number in between acquisition fields. Data between groups were compared using t tests that followed ANOVA when required. Statistical analyses were performed using SPSS 20.

Western Blotting

Cells were lysed with RIPA buffer (Sigma) supplemented with SIGMAFAST Protease Inhibitor Tablets (Millipore-Sigma). Protein concentrations were determined using a Pierce BCA Protein Assay Kit (Thermo Fisher). Approximately 15 μg of protein was loaded per well in Mini-PROTEAN TGX Stain-Free Precast Gels (Bio-Rad). Gels were run at 150 V for approximately 75 min and then transferred to a nitrocellulose membrane using a Trans-Blot Turbo Transfer System (Bio-Rad). Membranes were blocked in 4% non-fat milk dissolved in Tris-buffered saline plus Tween 20 (TBST) buffer (Sigma-Aldrich) for 20 min, then incubated with primary antibodies overnight at 4°C with shaking. Blots were washed three times in TBST for 5 min and then incubated with appropriate mouse or rabbit secondary antibodies for 1 hr at room temperature. Blots were washed a further three times in TBST for 5 min, then imaged using a ChemiDoc XRS+ System (Bio-Rad). Blots were imaged and analyzed using ImageLab software, and statistical analysis was performed using Student's t tests when two sample conditions were present and one-way ANOVA when more than two sample conditions were present. Further details on the antibodies used can be found in Supplemental Experimental Procedures.

Pharmacological Blockade

NPCs were plated on glass coverslips coated with Matrigel, and differentiated in medium supplemented with 10 μM and 50 μM of APV (Tocis) or 3 μM of ifenprodil (Tocris) for 30 days. All other cell-culture parameters were identical to the differentiation protocol cited above. Every 3 days, half of the medium was exchanged. After 30 days the coverslips were removed and prepared for ICC as described above.

Additional Methods

Sanger sequencing, RNA extraction and sequencing, comparison of transcriptomics profiles, and GEO analysis can be found in Supplemental Experimental Procedures.

ACCESSION NUMBERS

Both raw and analyzed data have been uploaded to the GEO database under the study number GEO: GSE114685.

SUPPLEMENTAL INFORMATION

Supplemental Information includes Supplemental Experimental Procedures, five figures, two tables, and six videos can be found with this article online at https://doi.org/10.1016/j.stemcr.2018.05.018.

AUTHOR CONTRIBUTIONS

G.M., S.B., H.P., M.J., P.H., H.W., H.S., C.B.-P., S.G.T.-P., and C.E. generated primary cell-culture data. J.-F.T. performed bioinformatics analyses. E.G. and V.G. grew and collected patient somatic cells. G.T., N.M., and C.E. provided reagents and support. V.S., G.M., T.M.D., and E.A.F. generated Ca^{2+} imaging videos. L.A.O. drew illustrative figures. S.B., G.M., and C.E. wrote the manuscript.

ACKNOWLEDGMENTS

Dr. Barbara Burton and Dr. Joel Charrow provided samples from patient E413G to V.G. This work was supported by the Canadian Institutes of Health Research (CIHR) under grant number PJT-153271. C.E. is funded by grants from the Canadian Institute of Health Research and a Canada Research Chair award. S.B. is funded by an FRQS award; M.J. is funded by the Indonesian government; P.H. is funded by the Central government of China. Images were taken at the Molecular and Cellular Microscopy Platform at the Douglas Hospital Research Center. Melina Jaramillo Garcia helped set up the imaging experiments and the analysis. We are grateful to Keith Murai for helpful comments on the manuscript and for support from the Sandra and Alain Bouchard Intellectual Disabilities platform.

Received: October 3, 2017
Revised: May 25, 2018
Accepted: May 28, 2018
Published: June 21, 2018

REFERENCES

Aamodt, S.M., and Constantine-Paton, M. (1999). The role of neural activity in synaptic development and its implications for adult brain function. Adv. Neurol. *79*, 133–144.

Adams, D.R., Yuan, H., Holyoak, T., Arajs, K.H., Hakimi, P., Markello, T.C., Wolfe, L.A., Vilboux, T., Burton, B.K., Fajardo, K.F.,

et al. (2014). Three rare diseases in one Sib pair: RAI1, PCK1, GRIN2B mutations associated with Smith-Magenis syndrome, cytosolic PEPCK deficiency and NMDA receptor glutamate insensitivity. Mol. Genet. Metab. *113*, 161–170.

Bading, H., Ginty, D.D., and Greenberg, M.E. (1993). Regulation of gene expression in hippocampal neurons by distinct calcium signaling pathways. Science *260*, 181–186.

Balazs, R., Hack, N., and Jorgensen, O.S. (1988). Stimulation of the N-methyl-D-aspartate receptor has a trophic effect on differentiating cerebellar granule cells. Neurosci. Lett. *87*, 80–86.

Behar, T.N., Scott, C.A., Greene, C.L., Wen, X., Smith, S.V., Maric, D., Liu, Q.Y., Colton, C.A., and Barker, J.L. (1999). Glutamate acting at NMDA receptors stimulates embryonic cortical neuronal migration. J. Neurosci. *19*, 4449–4461.

Bell, S., Peng, H., Crapper, L., Kolobova, I., Maussion, G., Vasuta, C., Yerko, V., Wong, T.P., and Ernst, C. (2017). A rapid pipeline to model rare neurodevelopmental disorders with simultaneous CRISPR/Cas9 gene editing. Stem Cells Transl. Med. *6*, 886–896.

Blanton, M.G., Lo Turco, J.J., and Kriegstein, A.R. (1990). Endogenous neurotransmitter activates N-methyl-D-aspartate receptors on differentiating neurons in embryonic cortex. Proc. Natl. Acad. Sci. USA *87*, 8027–8030.

Bliss, T.V., and Collingridge, G.L. (1993). A synaptic model of memory: long-term potentiation in the hippocampus. Nature *361*, 31–39.

Bourgeron, T. (2015). From the genetic architecture to synaptic plasticity in autism spectrum disorder. Nat. Rev. Neurosci. *16*, 551–563.

Brenneman, D.E., Forsythe, I.D., Nicol, T., and Nelson, P.G. (1990). N-methyl-D-aspartate receptors influence neuronal survival in developing spinal cord cultures. Brain Res. Dev. Brain Res. *51*, 63–68.

Cohen, S., and Greenberg, M.E. (2008). Communication between the synapse and the nucleus in neuronal development, plasticity, and disease. Annu. Rev. Cell Dev. Biol. *24*, 183–209.

Cull-Candy, S., Brickley, S., and Farrant, M. (2001). NMDA receptor subunits: diversity, development and disease. Curr. Opin. Neurobiol. *11*, 327–335.

Das, S., Sasaki, Y.F., Rothe, T., Premkumar, L.S., Takasu, M., Crandall, J.E., Dikkes, P., Conner, D.A., Rayudu, P.V., Cheung, W., et al. (1998). Increased NMDA current and spine density in mice lacking the NMDA receptor subunit NR3A. Nature *393*, 377–381.

Dimassi, S., Andrieux, J., Labalme, A., Lesca, G., Cordier, M.P., Boute, O., Neut, D., Edery, P., Sanlaville, D., and Schluth-Bolard, C. (2013). Interstitial 12p13.1 deletion involving GRIN2B in three patients with intellectual disability. Am. J. Med. Genet. A *161A*, 2564–2569.

Endele, S., Rosenberger, G., Geider, K., Popp, B., Tamer, C., Stefanova, I., Milh, M., Kortum, F., Fritsch, A., Pientka, F.K., et al. (2010). Mutations in GRIN2A and GRIN2B encoding regulatory subunits of NMDA receptors cause variable neurodevelopmental phenotypes. Nat. Genet. *42*, 1021–1026.

Ernst, C. (2016). Proliferation and differentiation deficits are a major convergence point for neurodevelopmental disorders. Trends Neurosci. *39*, 290–299.

Freunscht, I., Popp, B., Blank, R., Endele, S., Moog, U., Petri, H., Prott, E.C., Reis, A., Rubo, J., Zabel, B., et al. (2013). Behavioral phenotype in five individuals with de novo mutations within the GRIN2B gene. Behav. Brain Funct. *9*, 20.

Hu, C., Chen, W., Myers, S.J., Yuan, H., and Traynelis, S.F. (2016). Human GRIN2B variants in neurodevelopmental disorders. J. Pharmacol. Sci. *132*, 115–121.

Kim, H.G., Kishikawa, S., Higgins, A.W., Seong, I.S., Donovan, D.J., Shen, Y., Lally, E., Weiss, L.A., Najm, J., Kutsche, K., et al. (2008). Disruption of neurexin 1 associated with autism spectrum disorder. Am. J. Hum. Genet. *82*, 199–207.

Kutsuwada, T., Sakimura, K., Manabe, T., Takayama, C., Katakura, N., Kushiya, E., Natsume, R., Watanabe, M., Inoue, Y., Yagi, T., et al. (1996). Impairment of suckling response, trigeminal neuronal pattern formation, and hippocampal LTD in NMDA receptor epsilon 2 subunit mutant mice. Neuron *16*, 333–344.

Monteiro, P., and Feng, G. (2017). SHANK proteins: roles at the synapse and in autism spectrum disorder. Nat. Rev. Neurosci. *18*, 147–157.

Monyer, H., Burnashev, N., Laurie, D.J., Sakmann, B., and Seeburg, P.H. (1994). Developmental and regional expression in the rat brain and functional properties of four NMDA receptors. Neuron *12*, 529–540.

O'Roak, B.J., Deriziotis, P., Lee, C., Vives, L., Schwartz, J.J., Girirajan, S., Karakoc, E., Mackenzie, A.P., Ng, S.B., Baker, C., et al. (2011). Exome sequencing in sporadic autism spectrum disorders identifies severe de novo mutations. Nat. Genet. *43*, 585–589.

Platzer, K., Yuan, H., Schutz, H., Winschel, A., Chen, W., Hu, C., Kusumoto, H., Heyne, H.O., Helbig, K.L., Tang, S., et al. (2017). GRIN2B encephalopathy: novel findings on phenotype, variant clustering, functional consequences and treatment aspects. J. Med. Genet. *54*, 460–470.

Russell, R., Ilg, M., Lin, Q., Wu, G., Lechel, A., Bergmann, W., Eiseler, T., Linta, L., Kumar, P.P., Klingenstein, M., et al. (2015). A dynamic role of TBX3 in the pluripotency circuitry. Stem Cell Rep. *5*, 1155–1170.

Sala, C., Rudolph-Correia, S., and Sheng, M. (2000). Developmentally regulated NMDA receptor-dependent dephosphorylation of cAMP response element-binding protein (CREB) in hippocampal neurons. J. Neurosci. *20*, 3529–3536.

Salussolia, C.L., Prodromou, M.L., Borker, P., and Wollmuth, L.P. (2011). Arrangement of subunits in functional NMDA receptors. J. Neurosci. *31*, 11295–11304.

Sheng, M., Cummings, J., Roldan, L.A., Jan, Y.N., and Jan, L.Y. (1994). Changing subunit composition of heteromeric NMDA receptors during development of rat cortex. Nature *368*, 144–147.

Tovar, K.R., and Westbrook, G.L. (1999). The incorporation of NMDA receptors with a distinct subunit composition at nascent hippocampal synapses in vitro. J. Neurosci. *19*, 4180–4188.

Vaccarino, F.M., Hayward, M.D., Nestler, E.J., Duman, R.S., and Tallman, J.F. (1992). Differential induction of immediate early genes by excitatory amino acid receptor types in primary cultures of cortical and striatal neurons. Brain Res. Mol. Brain Res. *12*, 233–241.

Williams, K. (1993). Ifenprodil discriminates subtypes of the N-methyl-D-aspartate receptor: selectivity and mechanisms at recombinant heteromeric receptors. Mol. Pharmacol. *44*, 851–859.

Williams, K., Russell, S.L., Shen, Y.M., and Molinoff, P.B. (1993). Developmental switch in the expression of NMDA receptors occurs in vivo and in vitro. Neuron *10*, 267–278.

Xia, Z., Dudek, H., Miranti, C.K., and Greenberg, M.E. (1996). Calcium influx via the NMDA receptor induces immediate early gene transcription by a MAP kinase/ERK-dependent mechanism. J. Neurosci. *16*, 5425–5436.

Stem Cell Reports, Volume *11*

Supplemental Information

Disruption of *GRIN2B* Impairs Differentiation in Human Neurons

Scott Bell, Gilles Maussion, Malvin Jefri, Huashan Peng, Jean-Francois Theroux, Heika Silveira, Vincent Soubannier, Hanrong Wu, Peng Hu, Ekaterina Galat, S. Gabriela Torres-Platas, Camille Boudreau-Pinsonneault, Liam A. O'Leary, Vasiliy Galat, Gustavo Turecki, Thomas M. Durcan, Edward A. Fon, Naguib Mechawar, and Carl Ernst

Supplementary Figures

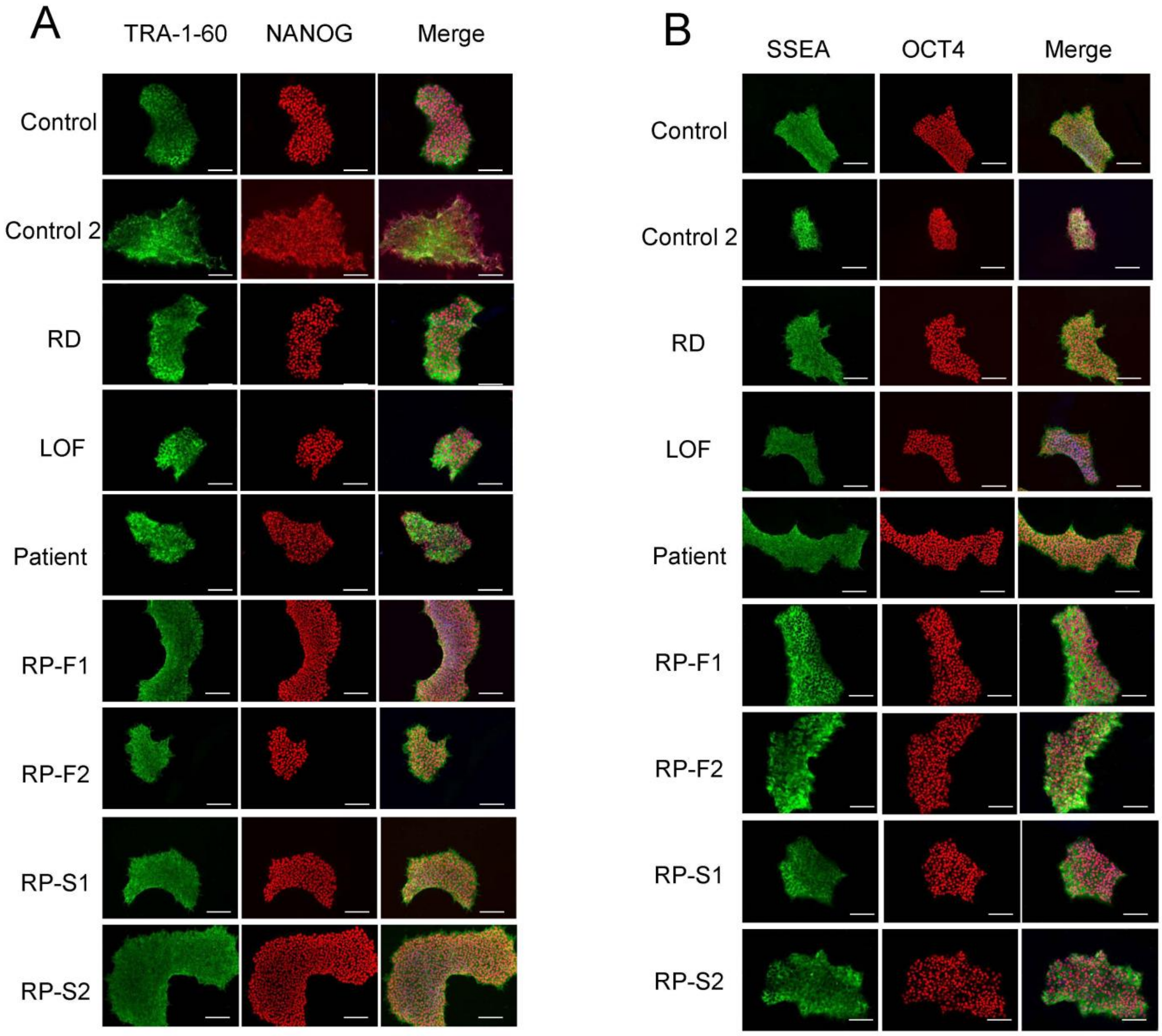

Supplementary Figure 1. Quality Control staining for pluripotency of cell lines used in this study. Related to Figure 1

Staining of the pluripotent markers TRA-1-60 and NANOG (shown in A) and SSEA and OCT4 (shown in B) from all lines used in this study. Scale bar represents 50µm. Select images can be found in Figure 1.

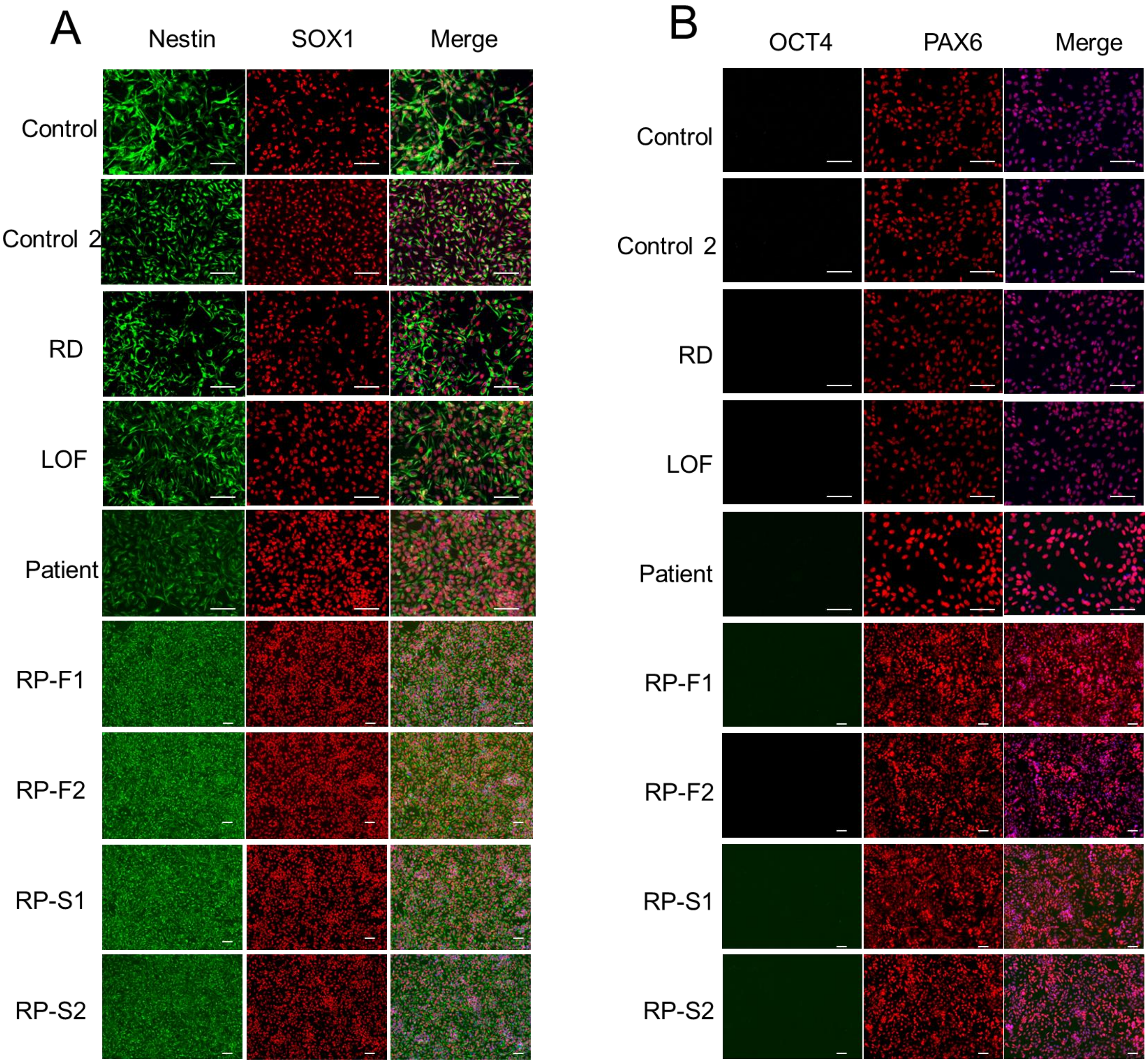

Supplementary Figure 2. Quality Control staining for neural progenitor cells. Related to Figure 1

Immunostaining of Nestin and SOX1 (shown in A), and OCT4 and PAX6 (shown in B) for all cell lines used in this study. Scale bar represents 50µm. Select images can be found in Figure 1. A representative image from A) can be found in Figure 2.

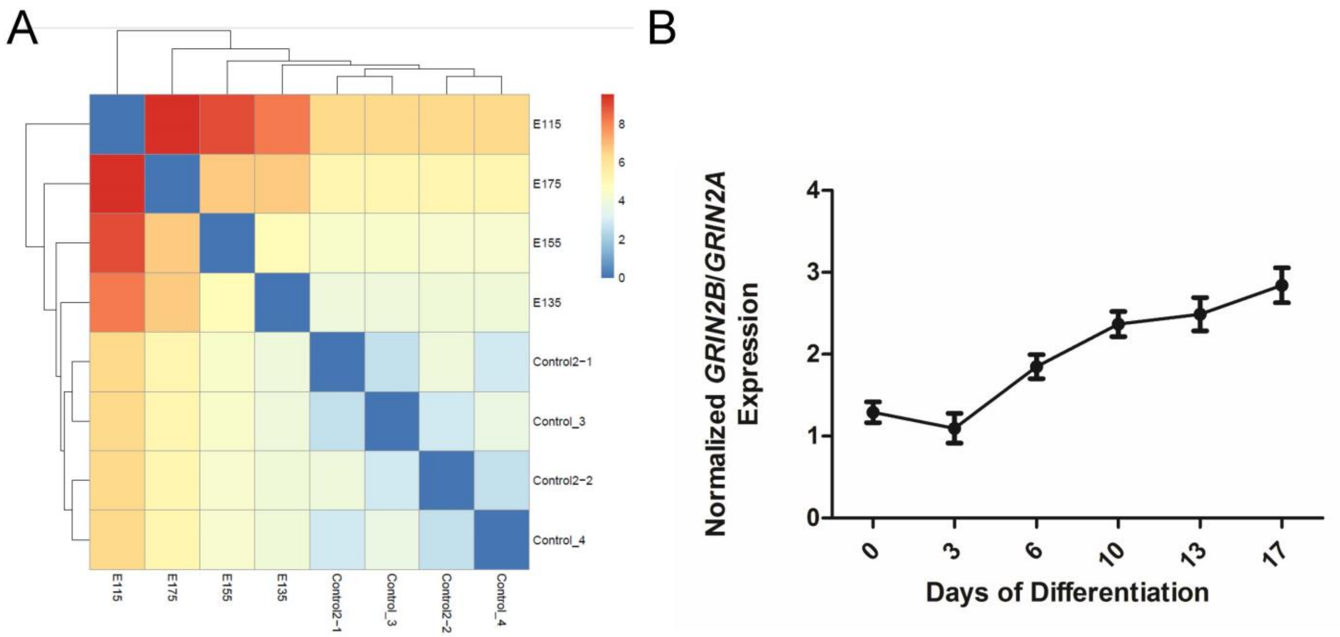

Supplementary Figure 3. Characterization of forebrain neurons. Related to Figure 1

A) Clustering of RNA-Seq data from D30 forebrain neurons with RNA-SEQ data from mouse radial precursor single cell expression profiles at four different timepoints. The Gene matrix was normalized using a regularized log transformation. Mouse radial precursor expression profiles were obtained from Yuzwa et al. (2017).
B) Ratio of GRIN2B/ GRIN2A expression during neuronal development ranging from 0 to 18 days after the initiation of differentiation from NPCs.

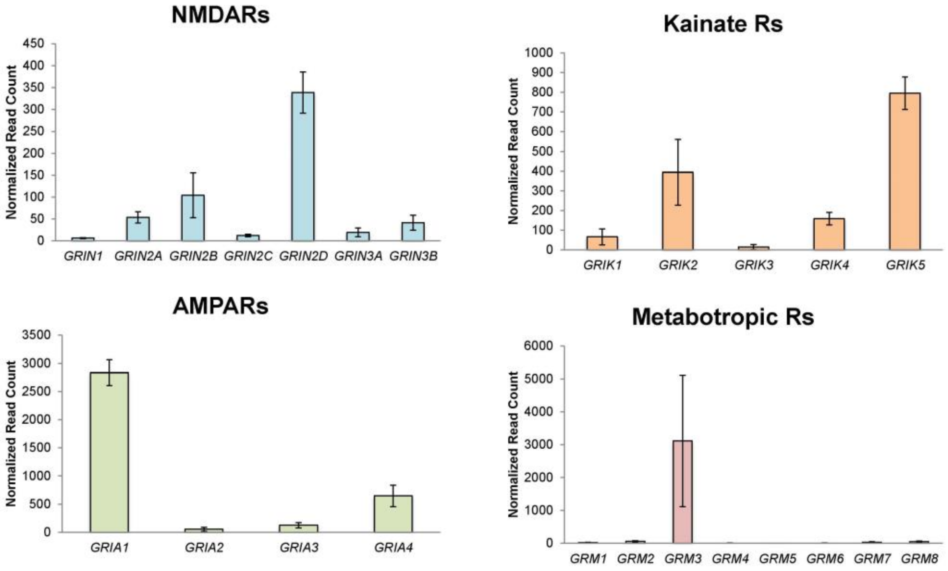

Supplementary Figure 4. Profile of NMDA, AMPA, Kainate, and Metabotropic receptor genes in control NPCs. Related to Figure 2

RNA sequencing reads for all NMDA, AMPA, Kainate and Metabotropic receptor genes. Reads normalized using deseq2 normalization algorithm.

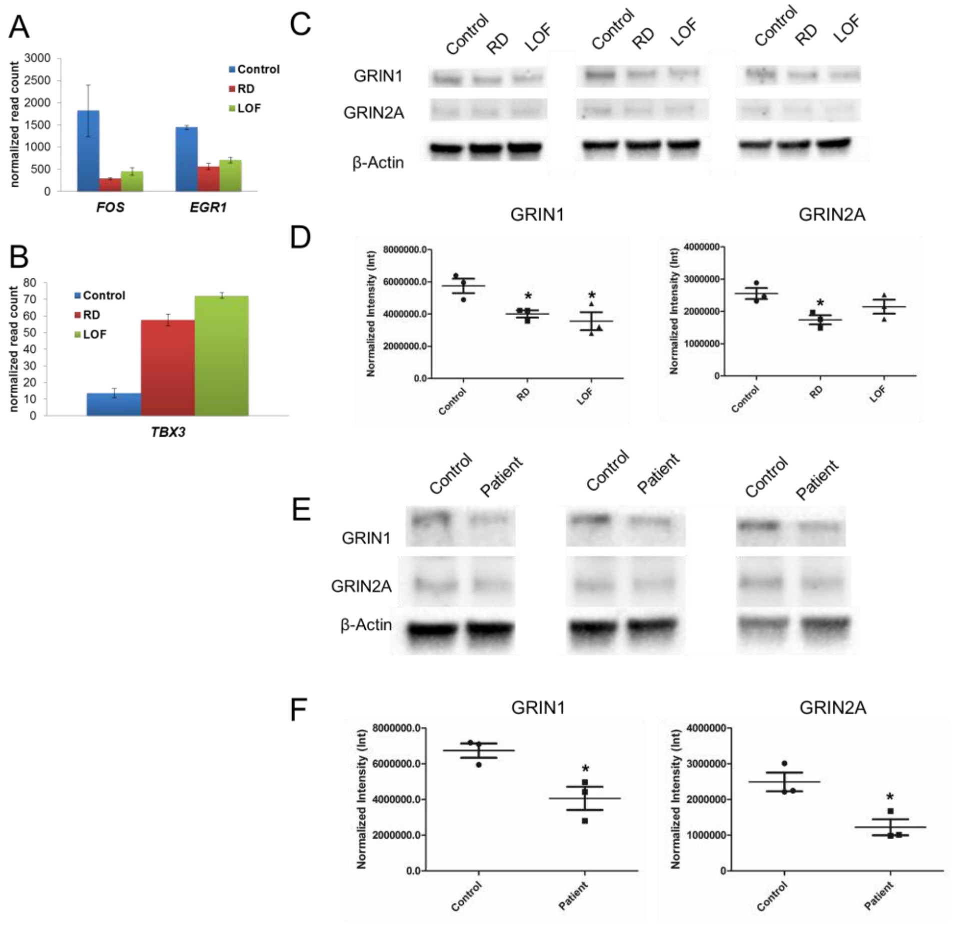

Supplementary Figure 5. Deficiency in *GRIN2B* expression is correlated with decreased expression of *GRIN1* and *GRIN2A*. Related to Figure 3 and Figure 4

A) Immediate early genes FOS and EGR1 show reduced expression in *GRIN2B* deficiency models (RNAseq data), consistent with loss of NMDA signalling

B) Increased TBX3 expression in GRIN2B deficiency models is consistent with cells in a more proliferative state.

C) Independent triplicate Western blots of GRIN1, GRIN2A and β-Actin using lysates from control, RD, and LOF neurons taken after four weeks of differentiation from NPCs (Day=30).

D) Quantification of the Western blots shown in A for GRIN1 and GRIN2A, using β-Actin for normalization. *: $p<0.05$

E) Independent, triplicate Western blots of GRIN1, GRIN2A and β-Actin using lysates from patient and control neurons taken at D=28.

F) Quantification of the Western blots shown in B for GRIN1 and GRIN2A, using β-Actin for normalization. *: $p<0.05$

Supplementary Tables

Supplemental Table 1. Cell lines used in this study. Name, source of the cell line, the sex, age, ethnicity characteristics, reprogramming method and gene editing is listed for each line. Related to Figure 1

Name	Source	Sex	Age	Ethnicity	Clinical Characteristics	Reprogramming Method	Gene Editing Method
Control 1	Coriell (GM07492)	M	17	Caucasian	Healthy	Episomal	N/A
Control 2	Patient Biopsy	M	21	Caucasian	Healthy	Episomal	N/A
Patient	Patient Biopsy	F	5	Caucasian	Delayed development, intellectual disability, hypotonia	Episomal	N/A
RD	Control 1	N/A	N/A	N/A	N/A	Episomal	CRISPR/ $CAS9^{wt}$
LOF	Control 1	N/A	N/A	N/A	N/A	Episomal	CRISPR/ $CAS9^{wt}$
RP-F1	Patient	N/A	N/A	N/A	N/A	Episomal	CRISPR/ $CAS9^{D10A}$ Nickase
RP-F2	Patient	N/A	N/A	N/A	N/A	Episomal	CRISPR/ $CAS9^{D10A}$ Nickase
RP-S1	Patient	N/A	N/A	N/A	N/A	Episomal	CRISPR/ $CAS9^{D10A}$ Nickase
RP-S2	Patient	N/A	N/A	N/A	N/A	Episomal	CRISPR/ $CAS9^{D10A}$ Nickase

Supplemental Table 2: Cell lines and replicates used in this study. The cell lines and number of replicates used in each Figure is listed. Related to Experimental Procedures

Experimental Figure	Cell lines Used	Replicates (n)
Figure 1E	Control 1	8
Figure 2G	Control 1	≥46
Figure 3G	Control 1, LOF, RD	3
Figure 3I	Control 1, LOF, RD	8
Figure 4D	Control 1, Control 2, Patient	3
Figure 4F	Control 1, Patient	7
Figure 4K	RP-F1, RP-F2, RP-S1, RP-S2	6
Figure 5C	Control 1	7
Figure 6B	Control 1, RD, LOF, Patient	≥58
Supplementary Figure 3B	Control 2	3
Supplementary Figure 4	Control 1	
Supplementary Figure 5A	Control 1, LOF, RD	3
Supplementary Figure 5B	Control 1, LOF, RD	3
Supplementary Figure 5D	Control 1, LOF, RD	3
Supplementary Figure 5F	Control 1, Patient	3

Supplemental Experimental Procedures

Sanger sequencing

DNA was extracted from iPSCs using a QIAamp DNA Mini Kit (QIAGEN). Amplification of putative CRISPR KO and repair colonies was performed in 25 µl reaction volume consisting of 10 µL nuclease free water, 12.5 µL Taq green master mix, 0.5 µL forward primer, 0.5 µL reverse primer, 0.5 µL DMSO, 1µL template. PCR was performed using a S1000™ Thermal Cycler (BioRad). PCR products were examined by electrophoresis at 100 V for 30 min in a 1.5% (w/v) agarose gel in 1 x TAE buffer to confirm product purity, and then shipped to Genome Quebec (Montreal, Canada) and sequenced on a using a 3730xl DNA Analyzer (Illumina). Primer sequences used to confirm gene editing can be found in CRISPR supplementary materials

RNA extraction and quality control

Cells were washed with PBS and detached using 0.05% trypsin-EDTA, and resuspended in Qiazol (Qiagen). RNA was extracted using an miRNeasy kit (Qiagen). Prior to RNA sequencing, appropriate RNA concentration, 260/230 and 260/280 ratios were determined by using a NanoDrop 2000 UV-Vis Spectrophotometer (Nanodrop). The quality of the RNA and the 28S/18S ratios were assessed using an Agilent 2100 Bio analyser (Aligent) and the RNA 6000 NanoChip (Aligent). All samples with RIN values below 9 were excluded from further analysis.

RNA-Sequencing

All libraries were prepared by expert technicians at the McGill University and Genome Quebec Innovation Center. Replicates for each cell line were grown in different T75 flasks, and extraction of RNA was done independently for each flask. Eight libraries were run per lane of an Illumina HiSeqV4 2500 flow cell (125 bp paired-end reads), which achieved an average of ~40 million reads per library. For bioinformatic processing, we used FASTX-Toolkit, TopHat Bowtie2, and Cufflinks2 with default parameters to preprocess, align, and assemble reads into transcripts, estimate abundance, and test differential expression.

Comparison of transcriptomics profiles

Mouse radial precursor single cell expression profiles at three different timepoints (embryonic days 11, 13, 15 and 17) were obtained from Yuzwa et al. (2017). Average expression profiles were computed on cortical cells for each developmental stage. In an effort to compare our RNASeq read counts with their Single Cell transcript counts, we divided each gene's read counts by its transcript's size. We selected a number of common genes present in our RNASeq and their 4 timepoints. Correspondence between our human genes and their mouse genes was established based on gene

symbols. The resulting dataset is composed of 4 average expression profiles (one for each timepoint) and 4 RNASeq Control samples from our experiment (composed of two batches of two samples) over a total of 11869 genes. The gene matrix was normalized using a regularized log transformation in DESeq2. Limma's removeBatchEffect algorithm was applied to the data to reduce the impact of the 3 expected batches (data from Yuzwa et al. and the two batches from our controls). Batch-corrected log transformed counts were then used for two hierarchical clustering experiments, respectively using distance and Pearson's R as dissimilarity/similarity metrics. Both raw and analyszed data were uploaded to the GEO database (https://www.ncbi.nlm.nih.gov/geo/) under the study number GSE114685.

GEO analysis

Genes were considered as signicantly differentially expressed if they displayed a Bonferroni corrected P-value below 0.05. Differentially expressed genes were analysed using DAVID annotation tools (https://david-d.ncifcrf.gov/). To determine functional enrichments in our sets of significant differentially expressed genes, the first three out five layers of annotations were selected from Gene Expression Omnibus classifications in the categories “biological process”, “cellular component” and “molecular function” A significant enrichment was considered in those categories for values below 0.05 after correction using a Bonferroni method.

Tables for Supplemental Experimental Procedures

Primers and probes used for qPCR analysis. Related to Experimental Procedures

Gene target	Reference
GAPDH	4310884E (Applied Biosystems)
GRIN2B	Hs00168230_m1 (Applied Biosystems)
GAPDH	Hs.PT.39a.22214836 (IDT)
MET	Hs.PT.58.339430 (IDT)
MKI67	Hs.PT.58.27920212 (IDT)

Antibodies used in immunocytochemistry. Concentration used, Supplier and Catalog number is provided for each antibody. Related to Experimental Procedures

Antibody	Concentration Used	Supplier	Catalog Number
Tuj1	1/2000	Abcam	ab14545
Nestin	1/2000	Stemcell Technologies	60091
SOX1	1/1000	Stemcell Technologies	60095
OCT4	1/100	Stemcell	60093

		Technologies	
PAX6	1/500	Stemcell Technologies	60094
TRA-1-60	1/100	Abcam	ab109884
Nanog	1/100	Abcam	ab109884
SSEA	1/100	Abcam	ab109884
VGLUT1	1/300	Abcam	ab77822
GABA	1/500	Abcam	ab86186
GFAP	1/500	Abcam	ab7260
S100B	1/200	Abcam	ab52642
MAP2	1/100	Abcam	ab109884
GRIN2B	1/250	Abcam	ab93610
KI67	1/500	Abcam	ab92742
MET	1/100	Abcam	ab51067
ALEXA 488	1/2000	Invitrogen	A-11008
ALEXA 555	1/2000	Invitrogen	A-21422

Antibodies used in western blotting. Concentration used, supplier, and catalog number is provided for each antibody. Related to Experimental Procedures

Protein Target	Concentration Used	Supplier	Catalog Number
GRIN2B	1/2000	Abcam	ab65783
GRIN2A	1/1000	Abcam	ab124913
GRIN1	1/1000	Abcam	ab109182
KI67	1/3000	Abcam	ab92742
MET	1/1000	Abcam	ab51067
β-actin	1/5000	Abcam	ab8227
C-FOS	1/1000	Abcam	ab190289
P-CREB (S133)	1/1000	Abcam	ab32096
CREB	1/1000	Abcam	ab32515
Rabbit Anti-Mouse IgG		Abcam	ab97046
Goat Anti-Rabbit IgG	1/5000	Abcam	ab6721

Supplementary Information about *GRIN2B* KO CRISPR Experiment (Related to Figure 3)

Section of *GRIN2B* Gene targeted: hg19_dna range=chr12:13722703-13722942

TGATGTTTTGGACTGGCCATCAGTAGAGGACAAATGGGCACTTTCCCTTTCTTGAA
CTCACCATCTCCAAAGAGCTGCAGGATAGCAAGGTCCACCTGGCGCTTCCACCCA
GAATCTTTTTGGATGGCAATGCCATAGCCAGTGGAAGCAAAGACCTTCCCACTGCC
AATGGTCACCAGCTTGCAGCCTTCATCTCTGCCTGCCATATAGTTCAGCACTGCTG
CATCATAGATGAAGGCA

FWD CRIPSR gRNA sequence:

GATGGCAATGCCATAGCCAGTGG

REV CRISPR gRNA sequence:

GGACCGCGAAGGTGGGTCTTAGA

Sanger Forward Primer sequence: TTTGGACGTGGCCATCAGTAG

Sanger Reverse Primer sequence: TATATGGCAGGCAGAGATGAAG

Gel of PCR products for Sanger Sequencing

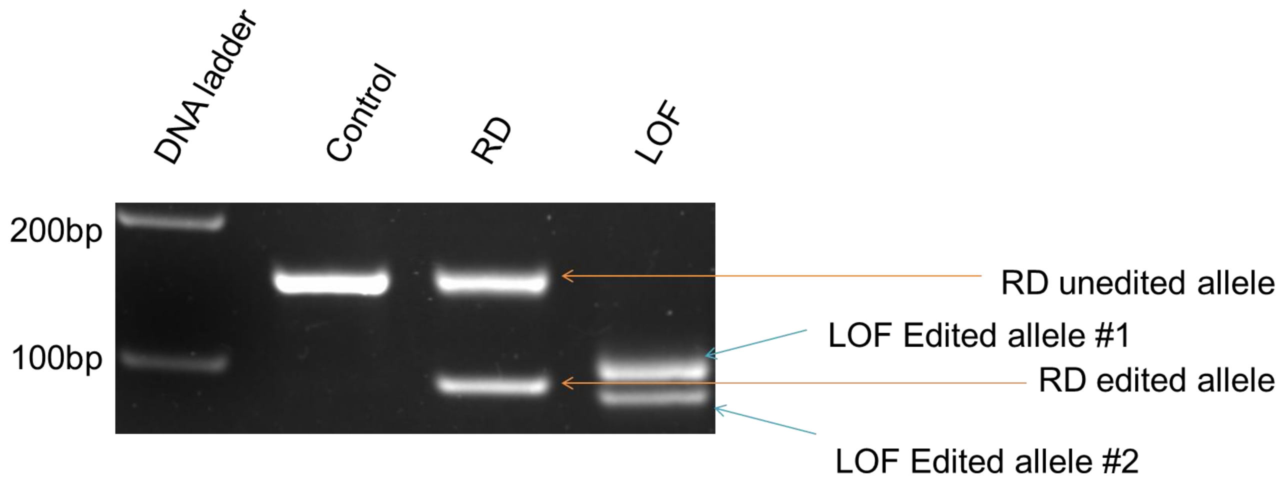

Fasta sequences from Sanger sequencing

Control

ttctgaagaagtgagttttggatggcctcagtagaggacaaatgggcactttccctttcttgaactcaccatctccaaagagct
gcaggatagcaaggtccacctggcgcttccacccagaatctttttggatggcaatgccatagccagtggaagcaaagac
cttcccactgccaatggtcaccagcttgcagccttcatctctgcctgccatatagaa

RD High Band

ttctgaagaaggtgagttttggatggccatcagtagaggacaaatgggcactttccctttcttgaactcaccatctccaaaga
agctgcagggatagcaaggtccacctggcagctgtcccactgccaatggtcaccagcttgcagccgtcatctctacctgcc
atatagaa

RD Low Band

Nngggaagaagtgagttttggatggccatcagtagaggaaaatgggcactttccctttcttgaactcaccatctccaaaga
gctgcaggatagcaagaccttcccactgccaatggtcaccagcttgcagccttcatctctgcctgccatatagaa

LOF High Band

ttctgaaagagttgagttttggatggccatagtagaggacaaatgggcactttccctttcttgaactcaccatctccaaagag
ctgcaggatagcaaggtccacctggcgcttccacccagaatctttttggatggcaatgccatagccagtggaagcaaaga
ccttcccactgccaatggtcaccagcttgcagccttcatctctgcctgccatatagaa

LOF Low Band

cctggagaaggtgagttttggatggccatcagtagaggaaaatgggcactttccctttcttgaactcaccatctccaaagag
ctgcaggatagcaaggtccacctgccaatggtcaccagcttgcagccttcatctctgcctgccatatagaa

Alignment

```
Control         caaggtccacctggcgcttccacccagaatctttttggatggcaatgccatagccagtggaagcaaagaccttcccactgcca
RD High Band    caaggtccacctggcgcttccacccagaatctttttggatggcaatgccatagccagtggaagcaaagaccttcccactgcca
RD Low Band     caag----------------------------------------------------------------tccaaacatca
LOF High Band   caag--------------------------------------------------------------accttcccactgcca
LOF Low Band    caaggtccacctg-----------------------------------------------------------------cca
```

Chromatograms

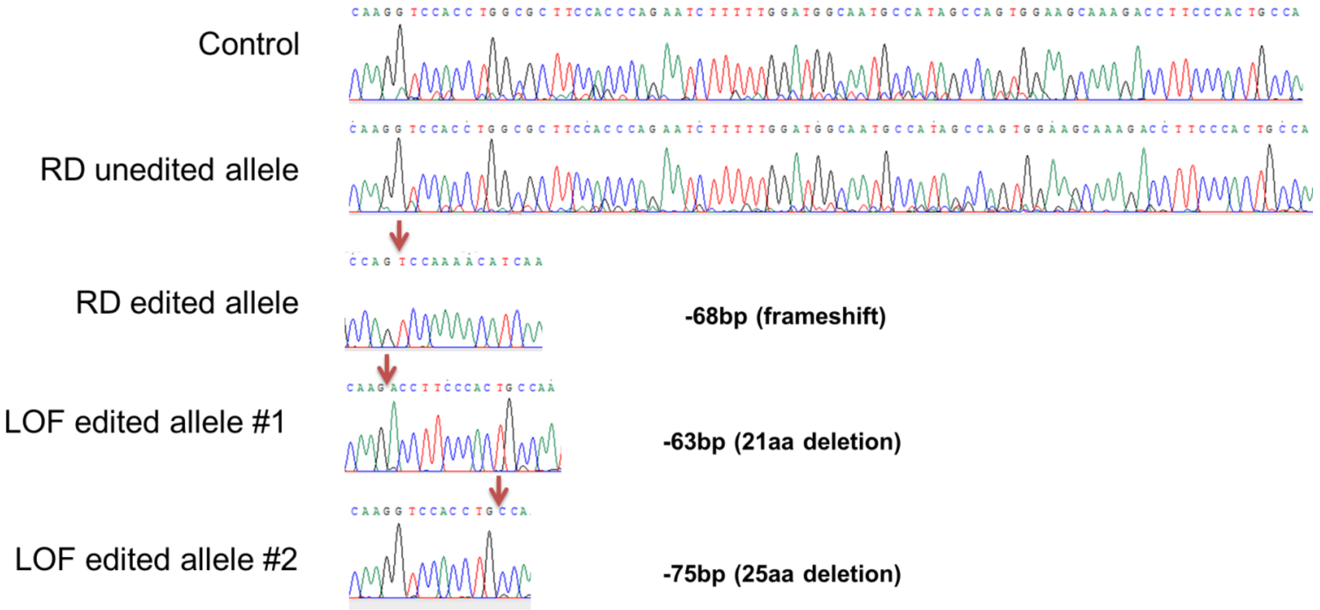

Supplementary Information about *GRIN2B* Repair CRISPR Experiment (related to Figure 4)

Point mutation sequence: (NM_000834.3:c.1238A>G)

Allele ID:152907

Variant type:single nucleotide variant

Cytogenetic location:12p13.1

Protein change: E413G

Patient Sequence: >hg19_dna range=chr12:13769238-13769704

TTTTGAGGGGAGGAATTTGTCCTTCAGTGAAGATGGCTACCAGATGCACCCGAAAC
TGGTGATAATTCTTCTGAACAAGGAGAGGAAGTGGGAAAGGGTGGGGAAGTGGAA
AGACAAGTCCCTGCAGATGAAGTACTATGTGTGGCCCCGAATGTGTCCAGAGACT
GAAGAGCAGGAGGATGACCATCTGAGCATTGTGACCCTGGAGGGGGCACCATTTG
TCATTGTGGAAAGTGTGGACCCTCTGAGTGGAACCTGCATGAGGAACACAGTCCC
CTGCCAAAAACGCATAGTCACTGAGAATAAAACAGACGAGGAGCCGGGTTACATC
AAAAAATGCTGCAAGGGGTTCTGTATTGACATCCTTAAGAAAATTTCTAAATCTGTG
AAGTTCACCTATGACCTTTACCTGGTTACCAATGGCAA

FWD CRISPR gRNA sequence: GAAAGTGTGGACCCTCTGAGTGG

REV CRISPR gRNA sequence: TGACAAATGGTGCCCCCTCCAGG

Repair Template FWD:
AATGTGTCCAGAGACTGAAGAGCAGGAGGATGACCATCTGAGCATTGTGACCCT GGAGGAGGCACCATTTGTCATTGTGGAAAGTGTGGACCCTCTGAGTGGAACCTG CATGAGGAACACAGTCCCCTGCCAAAAACGCATAGTCACTGAGA

Sanger Forward Primer sequence: GGGGAAGTGGAAAGACAAGT

Sanger Reverse Primer sequence: CAAAGCTGACTCTCCCATGC

Gel of PCR products for Sanger Sequencing

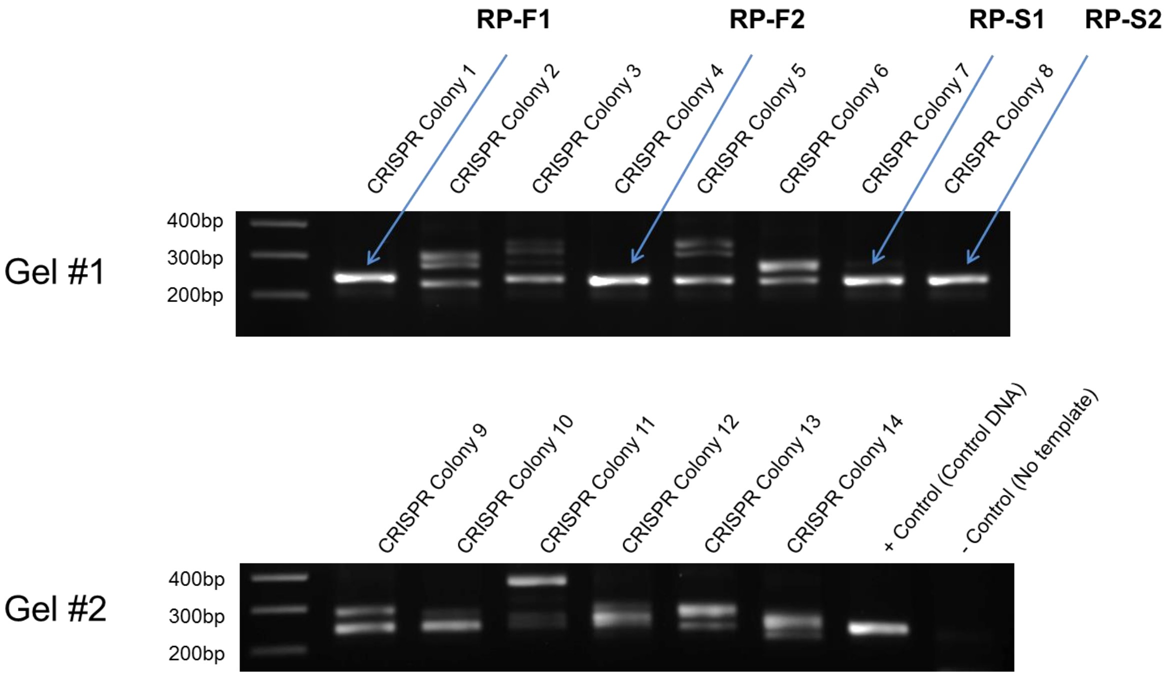

Fasta sequences from Sanger sequencing

Control

tggagagcctatcagtgactatcgtttttggcaggggactgtgttcctcatgcaggttccactcagagggtccacactttccac aatgacaaatggtgcctcctccagggtcacaatgctcagatggtcatcctcctgctcttcagtctctggacacattcggggcc acacatagtacttcatctgcagggacttgtctttccacttccccaa

Patient

tggagagcctatcagtgactatcgtttttggcaggggactgtgttcctcatgcaggttccactcagagggtccacactttccac aatgacaaatggtgcctcctccagggtcacaatgctcagatggtcatcctcctgctcttcagtctctggacacattcggggcc acacatagtacttcatctgcagggacttgtctttccacttccccaa

RP-F1

ggagagcctatcagtgactatcgtttttggcaggggactgtgttcctcatgcaggttccactcagagggtccacactttccac
aatgacaaatggtgcctcctccagggtcacaatgctcagatggtcatcctcctgctcttcagtctctggacacattcggggcc
acacatagtacttcatctgcagggacttgtctttccacttccccaa

RP-F2

ttggagagcctatcagtgactatcgtttttggcaggggactgtgttcctcatgcaggttccactcagagggtccacactttcca
caatgacaaatggtgcctcctccagggtcacaatgctcagatggtcatcctcctgctcttcagtctctggacacattcggggc
cacacatagtacttcatctgcagggacttgtctttccacttccccaa

RP-S1

tggagagcctaacagtgactatcgtttttggcaggggactgtgttcctcatgcaggttccactcagagggtccacactttcca
caatgacaaatggtgcctcctccagggtcacaatgctcagatggtcatcctcctgctcttcagtctctggacacattcggggc
cacacatagtacttcatctgcagggacttgtctttccacttccccaaa

RP-S2

Ttggagagcatatcagtgacatgcgtttttggcaggggactgtgttcctcatgcaggttccactcagagggtccacactttcc
acaatgacaaatggtgcctcctccagggtcacaatgctcagatggtcatcctcctgctcttcagtctctggacacattcgggg
ccacacatagtacttcatctgcagggacttgtctttccacttccccaa

Alignment (on reverse strand)

```
Control   CCACTCAGAGGGTCCACACTTTCCACAATGACAAATGGTGCCTCCTCCAGGGTCACAATGCTCAGATGGTCATCCT
Patient   CCACTCAGAGGGTCCACACTTTCCACAATGACAAATGGTGCCACCTCCAGGGTCACAATGCTCAGATGGTCATCCT
RP-F1     CCACTCAGAGGGTCCACACTTTCCACAATGACAAATGGTGCCACCTCCAGGGTCACAATGCTCAGATGGTCATCCT
RP-F2     CCACTCAGAGGGTCCACACTTTCCACAATGACAAATGGTGCCACCTCCAGGGTCACAATGCTCAGATGGTCATCCT
RP-S1     CCACTCAGAGGGTCCACACTTTCCACAATGACAAATGGTGCCTCCTCCAGGGTCACAATGCTCAGATGGTCATCCT
RP-S2     CCACTCAGAGGGTCCACACTTTCCACAATGACAAATGGTGCCTCCTCCAGGGTCACAATGCTCAGATGGTCATCCT
```

Chromatograms (on reverse strand)

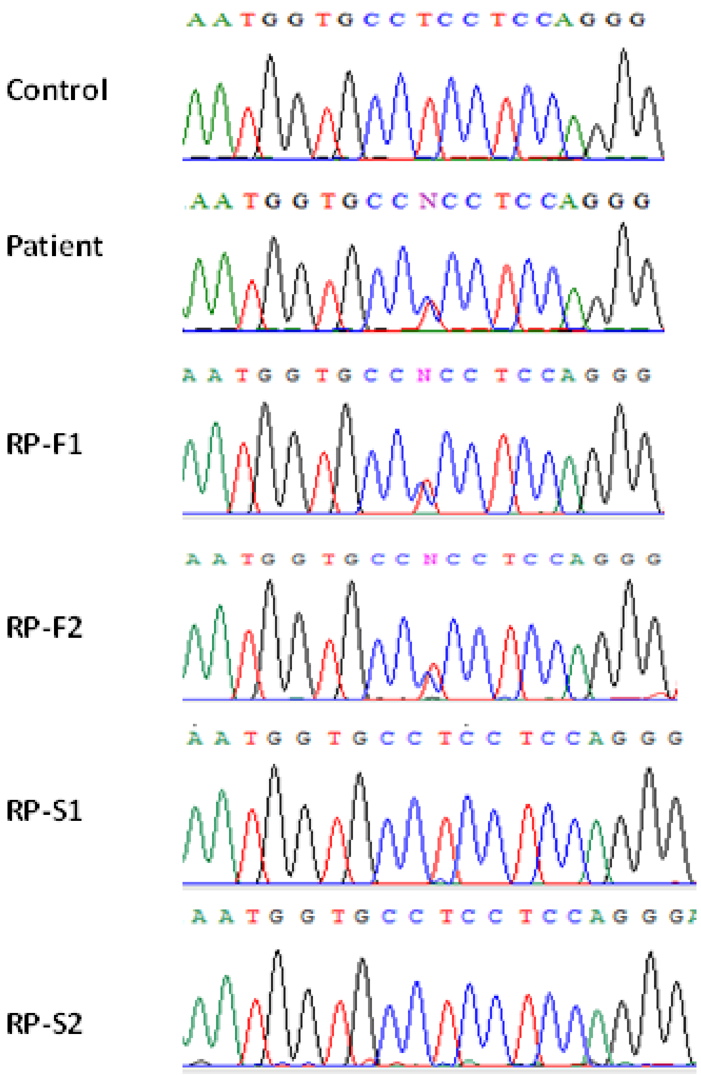

Supplement: Document S2. Article plus Supplemental Information [file mmc8.pdf]
